# Inducible apelin receptor knockdown reduces differentiation efficiency and contractility of hESC-derived cardiomyocytes

**DOI:** 10.1093/cvr/cvac065

**Published:** 2022-05-16

**Authors:** Robyn G C Macrae, Maria T Colzani, Thomas L Williams, Semih Bayraktar, Rhoda E Kuc, Anna L Pullinger, William G Bernard, Emma L Robinson, Emma E Davenport, Janet J Maguire, Sanjay Sinha, Anthony P Davenport

**Affiliations:** Experimental Medicine and Immunotherapeutics, University of Cambridge, Addenbrooke’s Hospital, Level 6, Addenbrooke’s Centre for Clinical Investigation, Box 110, Cambridge CB2 0QQ, UK; Wellcome-MRC Cambridge Stem Cell Institute, Jeffrey Cheah Biomedical Centre, University of Cambridge, Cambridge, UK; Wellcome-MRC Cambridge Stem Cell Institute, Jeffrey Cheah Biomedical Centre, University of Cambridge, Cambridge, UK; Experimental Medicine and Immunotherapeutics, University of Cambridge, Addenbrooke’s Hospital, Level 6, Addenbrooke’s Centre for Clinical Investigation, Box 110, Cambridge CB2 0QQ, UK; Wellcome-MRC Cambridge Stem Cell Institute, Jeffrey Cheah Biomedical Centre, University of Cambridge, Cambridge, UK; Experimental Medicine and Immunotherapeutics, University of Cambridge, Addenbrooke’s Hospital, Level 6, Addenbrooke’s Centre for Clinical Investigation, Box 110, Cambridge CB2 0QQ, UK; Experimental Medicine and Immunotherapeutics, University of Cambridge, Addenbrooke’s Hospital, Level 6, Addenbrooke’s Centre for Clinical Investigation, Box 110, Cambridge CB2 0QQ, UK; Wellcome-MRC Cambridge Stem Cell Institute, Jeffrey Cheah Biomedical Centre, University of Cambridge, Cambridge, UK; Wellcome-MRC Cambridge Stem Cell Institute, Jeffrey Cheah Biomedical Centre, University of Cambridge, Cambridge, UK; School of Medicine, Division of Cardiology, University of Colorado Denver, Aurora, CO, USA; Wellcome Sanger Institute, Cambridge, UK; Experimental Medicine and Immunotherapeutics, University of Cambridge, Addenbrooke’s Hospital, Level 6, Addenbrooke’s Centre for Clinical Investigation, Box 110, Cambridge CB2 0QQ, UK; Wellcome-MRC Cambridge Stem Cell Institute, Jeffrey Cheah Biomedical Centre, University of Cambridge, Cambridge, UK; Experimental Medicine and Immunotherapeutics, University of Cambridge, Addenbrooke’s Hospital, Level 6, Addenbrooke’s Centre for Clinical Investigation, Box 110, Cambridge CB2 0QQ, UK

**Keywords:** Apelin receptor, Stem cell, Cardiomyocyte, Cardiovascular disease

## Abstract

**Aims:**

The apelin receptor, a G protein-coupled receptor, has emerged as a key regulator of cardiovascular development, physiology, and disease. However, there is a lack of suitable human *in vitro* models to investigate the apelinergic system in cardiovascular cell types. For the first time we have used human embryonic stem cell-derived cardiomyocytes (hESC-CMs) and a novel inducible knockdown system to examine the role of the apelin receptor in both cardiomyocyte development and to determine the consequences of loss of apelin receptor function as a model of disease.

**Methods and results:**

Expression of the apelin receptor and its ligands in hESCs and hESC-CMs was determined. hESCs carrying a tetracycline-inducible short hairpin RNA targeting the apelin receptor were generated using the sOPTiKD system. Phenotypic assays characterized the consequences of either apelin receptor knockdown before hESC-CM differentiation (early knockdown) or in 3D engineered heart tissues as a disease model (late knockdown). hESC-CMs expressed the apelin signalling system at a similar level to the adult heart. Early apelin receptor knockdown decreased cardiomyocyte differentiation efficiency and prolonged voltage sensing, associated with asynchronous contraction. Late apelin receptor knockdown had detrimental consequences on 3D engineered heart tissue contractile properties, decreasing contractility and increasing stiffness.

**Conclusions:**

We have successfully knocked down the apelin receptor, using an inducible system, to demonstrate a key role in hESC-CM differentiation. Knockdown in 3D engineered heart tissues recapitulated the phenotype of apelin receptor down-regulation in a failing heart, providing a potential platform for modelling heart failure and testing novel therapeutic strategies.

## Graphical Abstract

**Graphical Abstract:**
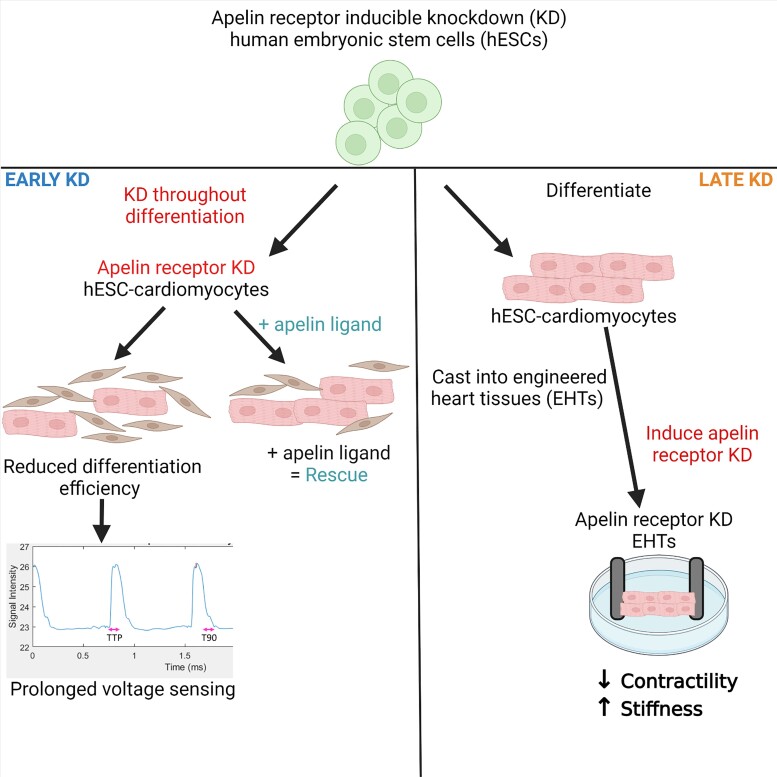
Effect of early or late apelin receptor inducible knock-down in cardiomyocytes derived from human embryonic stem cells

## 1. Introduction

The apelin system, a critical player in both cardiovascular development and (patho)physiology, consists of two ligands, apelin and elabela (ELA), activating a single G protein-coupled receptor (GPCR). The majority of apelin receptor knockout mice die *in utero*, displaying defective heart formation and poor vascularization.^1^ In contrast, apelin peptide knockout mice are viable and only develop cardiovascular phenotype with age.^2,3^ This discrepancy was explained by the discovery of the second peptide ligand for the receptor, ELA.^4,5^ Zebrafish embryo loss-of-function mutations in the ELA gene resulted in defective cardiac development, similar to that seen in apelin receptor knockout mice, which was rescued by the injection of apelin mRNA.

The apelin receptor is also an important mediator in cardiovascular physiology, promoting vasodilatation and positive inotropy, as well as in pathophysiology including heart failure, and pulmonary arterial hypertension.^6–12^ In a key study, Chen *et al.*^13^ reported that the apelin receptor gene was the most significantly increased of the ∼12 000 genes measured following implantation of a left ventricular assist device. Although there is an initial rise in circulating apelin, expression is reduced in later-stage heart failure. Crucially, apelin receptor expression is down-regulated to a lesser extent and remains responsive to its ligands. For example, infusion of apelin or ELA in animal models of heart failure has been shown to improve systolic and diastolic function, and reduce detrimental cardiac remodelling.^14–16^ Apelin receptor signalling has also been implicated in the regulation of both physiological and pathological organ fibrosis,^17^ and has been shown to reduce detrimental cardiac fibrosis and hypertrophy,^18^ which is a crucial step in heart failure progression.

Our aim was to generate, for the first time, an apelin receptor inducible knockdown system using a clinically relevant human embryonic stem cell-derived cardiomyocyte (hESC-CM) model. The objective was to recapitulate the reduction of the apelin receptor observed in heart failure patients. We first show these cells have a functioning apelin system, with similar expression levels of apelin receptor as native adult human cardiomyocytes. We used this system to address two fundamental questions: firstly, what is the effect of reduced apelin receptor expression from the onset of cardiomyocyte differentiation (early knockdown). Secondly, what is the effect of apelin receptor knockdown on the function of fully formed cardiomyocytes in a 3D engineered heart tissue (EHT) model (late knockdown).

We report that early apelin receptor knockdown throughout differentiation from hESC to hESC-CM, has a significant impact on phenotype reducing cardiomyocyte differentiation efficiency and increasing the number of fibroblasts, and functionally prolongs voltage signalling. Furthermore, we show that late knockdown of the apelin receptor following differentiation had detrimental effects on contractility in 3D engineered heart tissues, significantly decreasing force generation and increasing tissue stiffness, associated with increased collagen deposition. Together, these results support a pivotal role of the apelin receptor in cardiomyocyte development and physiological function, as well as having the potential for using this platform to screen for novel therapeutic agents for the treatment of heart failure.

## 2. Methods

For detailed methods see Supplementary material online, Materials.

### 2.1 Cell culture and hESC-CM differentiation

**Figure 1:**
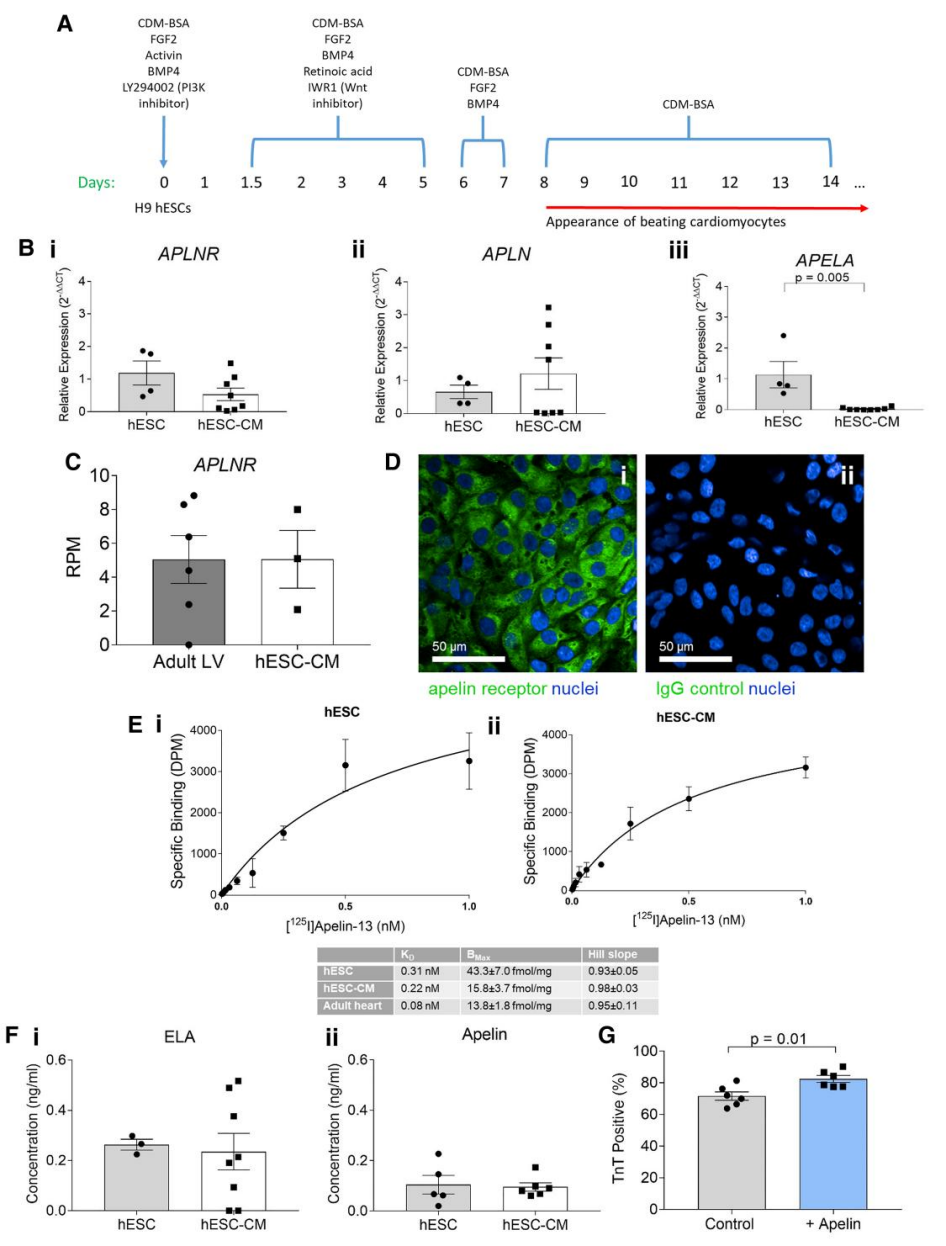
Human embryonic stem cells (hESCs) and hESC-derived cardiomyocytes (hESC-CMs) express the apelin receptor and its endogenous ligands. (*A*) Schematic representation of hESC to cardiomyocyte differentiation protocol. (*B*) Comparison of relative expression of (i) *APLNR*, (ii) *APLN*, and (iii) *APELA* in hESCs and hESC-derived cardiomyocytes determined by qRT-PCR. hESCs *n* = 4, hESC-derived cardiomyocytes *n* = 8. Expression displayed relative to mean hESC expression, means compared by unpaired, two-tailed Student’s *t*-test. For (iii) *P* = 0.005. (*C*) Comparison of reads per million (RPM) for *APLNR* mRNA in adult human left ventricle (LV, *n* = 6) and hESC-CMs (*n* = 3) by RNA-sequencing. Expression levels compared by unpaired, two-tailed Student’s *t*-test. (*D*) Representative fluorescent confocal images of endogenous expression of apelin receptor in hESC-CMs using an apelin receptor antibody (i), or hESC-CMs treated with an IgG isotype control (ii). Scale bars as indicated in figure. (*E*) Saturation radioligand binding in (i) hESCs and (ii) hESC-CMs using [^125^I]apelin-13, with measures of density and affinity compared with that of adult cardiomyocytes (inset table). (*F*) Comparison of concentration of (i) ELA and (ii) apelin peptides in conditioned supernatant from hESCs (*n* = 3 for ELA, *n* = 5 for apelin) and hESC-CMs (*n* = 8 for ELA, *n* = 6 for apelin). Means compared by unpaired, two-tailed Student’s *t*-test. (*G*) Cardiac troponin T (TnT) positive percentage from flow cytometric analysis of control hESC-CMs and hESC-CMs cultured in the presence of 10 nM [Pyr^1^]apelin-13 throughout differentiation (*n* = 4 for both). Means compared by unpaired, two-tailed Student’s *t*-test, *P* = 0.03. Data represent mean ± SEM.

H9 hESCs were maintained in culture as previously described^19^ and induced to differentiate to cardiomyocytes following a previously optimized protocol (*Figure 1A*) (adapted from Mendjan *et al.*^20^).

To determine the effect of apelin on hESC-CM differentiation, 10 nM [Pyr^1^]apelin-13 peptide was included in the culture media throughout differentiation, calculated from the equilibrium dissociation constant to occupy most of the receptors. [Pyr^1^]apelin-13 was used throughout as it is the predominant isoform in the human cardiovascular system,^21^ and ELA peptides are equipotent at the human apelin receptor.^22^

### 2.2 Real-time quantitative polymerase chain reaction

RNA extraction, reverse transcription, and real-time quantitative polymerase chain reaction (qRT-PCR) were performed as described in the Supplementary material online, *Materials*. Primer sequences are detailed in the Supplementary material online, *Materials*. Relative expression was calculated using the 2^(-ΔΔCT)^ method.^23^

### 2.3 Total RNA-sequencing and analysis

Surgical samples of control human heart tissue that were not suitable for transplantation were obtained from Royal Papworth Hospital Research Tissue Bank with informed consent and ethical approval (05/Q104/142). Bulk cell and tissue RNA-sequencing library preparation and analysis were performed as previously described^24,25^ and detailed in the Supplementary material online, *Materials*. All control human left vertical and hESC-CM RNA samples had RNA Integrity Numbers of 7.1–9.0 (7.8 ± 0.3).

### 2.4 Saturation and fixed concentration radioligand binding

Radiolabelled [^125^I]apelin-13 was used for saturation and fixed concentration binding studies as described previously.^26^

### 2.5 ELISA

Conditioned supernatant from hESCs and hESC-CMs was assayed for apelin and ELA production using commercially available ELISA kits.

### 2.6 Immunocytochemistry

Immunocytochemistry was performed as described in Supplementary material online, *Materials*, with details of the antibodies used also listed.

### 2.7 Generation of sOPTiKD apelin receptor knockdown system

An shRNA-based inducible apelin receptor knockdown system was generated using the sOPTiKD method.^27^ For full details, see Supplementary material online, *Materials*.

Early apelin receptor knockdown was induced by culturing hESCs with tetracycline for 4 days, maintaining tetracycline treatment throughout differentiation to cardiomyocyte and culturing resultant hESC-CMs in the presence of tetracycline. For late apelin receptor knockdown, upon completion of differentiation, hESC-CMs were cultured with tetracycline for 7 days. Apelin receptor knockdown efficiency was determined by qRT-PCR and saturation radioligand binding.

### 2.8 Early apelin receptor knockdown phenotypic assays

To quantify the effect of *APLNR* knockdown on hESC-CM differentiation efficiency, control and *APLNR* knockdown hESC-CMs were co-stained for the cardiac marker troponin T (TnT) and the fibroblast marker Thy-1, and analysed by flow cytometry. RNA-sequencing was performed and differential gene expression analysis was performed comparing control and early knockdown hESC-CMs. hESC-CMs were loaded with voltage sensitive and calcium-sensitive dyes, videos recorded and waveform time to peak and time to 90% decay extracted.

### 2.9 Apelin receptor knockdown engineered heart tissue generation

hESC-CMs were produced from the apelin receptor inducible knockdown hESCs without tetracycline inclusion, and combined in a collagen matrix with a supportive cell line (HS-27A) to produce 3D EHTs as previously described.^28,29^ EHTs were then cultured for 14 days to promote maturation, with tetracycline included in the culture medium throughout to induce *APLNR* knockdown.

### 2.10 Force transducer measurements

EHT force generation was determined by subjecting EHTs to stretch and electrical pacing, with a force transducer used to measure the resulting contractile response.

### 2.11 Collagen imaging

Fixed EHTs were imaged using second-harmonic imaging microscopy (SHIM) to determine collagen deposition.^30^

### 2.12 Declaration

Surgical samples of human tissue were obtained with informed consent from Royal Papworth Hospital Research Tissue Bank and ethical approval (05/Q104/142) as anonymized samples, according to the principles outlined in the Declaration of Helsinki. The hESC lines used were approved by the United Kingdom Stem Cell Bank and the use of these lines for the research was approved by the Steering Committee of the UKSCB.

### 2.13 Data analysis and statistics

All data are represented as mean ± SEM. Independent replicates for hESCs are defined as cells from distinct passages and for cardiomyocytes are defined as cells generated from distinct differentiations. The *n* values are stated in the figure legends. Statistical unpaired, two-tailed Student’s *t*-tests, or one-way ANOVA followed by Tukey’s *post hoc* test were performed and a *P*-value < 0.05 was considered as significant.

## 3. Results

### 3.1 Human undifferentiated ESCs and derived cardiomyocytes express the apelin receptor and its endogenous ligands

Genes encoding the apelin receptor and its endogenous ligands (*APLNR*, *APLN*, and *APELA*) were expressed in undifferentiated hESCs (*Figure 1B*). hESCs when differentiated into cardiomyocytes (*Figure 1A*), expressed standard cardiac markers at the gene and protein level, with a differentiation efficiency ∼70–80% as determined by positive cardiac TnT staining (see Supplementary material online, *Figure S1*). hESC-CMs retained apelin receptor gene expression. Similar levels of *APLNR* expression were found by RNA-sequencing analysis of hESC-CMs and adult human left ventricle (*Figure 1C*). hESC-CMs expressed low levels of the genes encoding both peptides (*APLN*, *APELA*) comparable to adult cardiomyocytes where little or no expression was detected (*Figure 1B*). Note that, whilst relative expression was low in four replicates, *APLN* mRNA was detectable in all eight replicates.

Differentiated hESC-CMs stained positively for apelin receptor protein (*Figure 1D*), indicating protein expression. [^125^I]apelin-13 binding was saturable, and bound with the expected subnanomolar affinity (*K*_D_) in hESCs and hESC-CMs (*Figure 1E*). Receptor density (*B*_Max_) was comparable to that in human adult cardiomyocytes. Hill slopes were close to unity for both hESCs and hESC-CMs, consistent with a one-site fit model of the apelin binding with a single affinity.

Importantly, hESCs and hESC-CMs consistently expressed both apelin and ELA peptides (0.26 ± 0.02 ng/mL ELA and 0.10 ± 0.04 ng/mL apelin in hESCs and 0.24 ± 0.07 ng/mL ELA and 0.10 ± 0.02 ng/mL apelin in hESC-CMs, *Figure 1F*), as determined by ELISA. Interestingly, expression of ELA was significantly higher than apelin in hESCs (*P* < 0.05).

We next tested whether hESC-CMs would respond to exogenous application of apelin ligands. [Pyr^1^]apelin-13 (10 nM) significantly increased TnT positive percentage from 71.7%±1.1 to 82.5%±0.9 (*Figure 1G*), indicating increased cardiomyocyte differentiation efficiency.

### 3.2 Significant apelin receptor knockdown can be induced in undifferentiated hESCs, early knockdown hESC-CMs, and in late knockdown hESC-CMs

**Figure 2:**
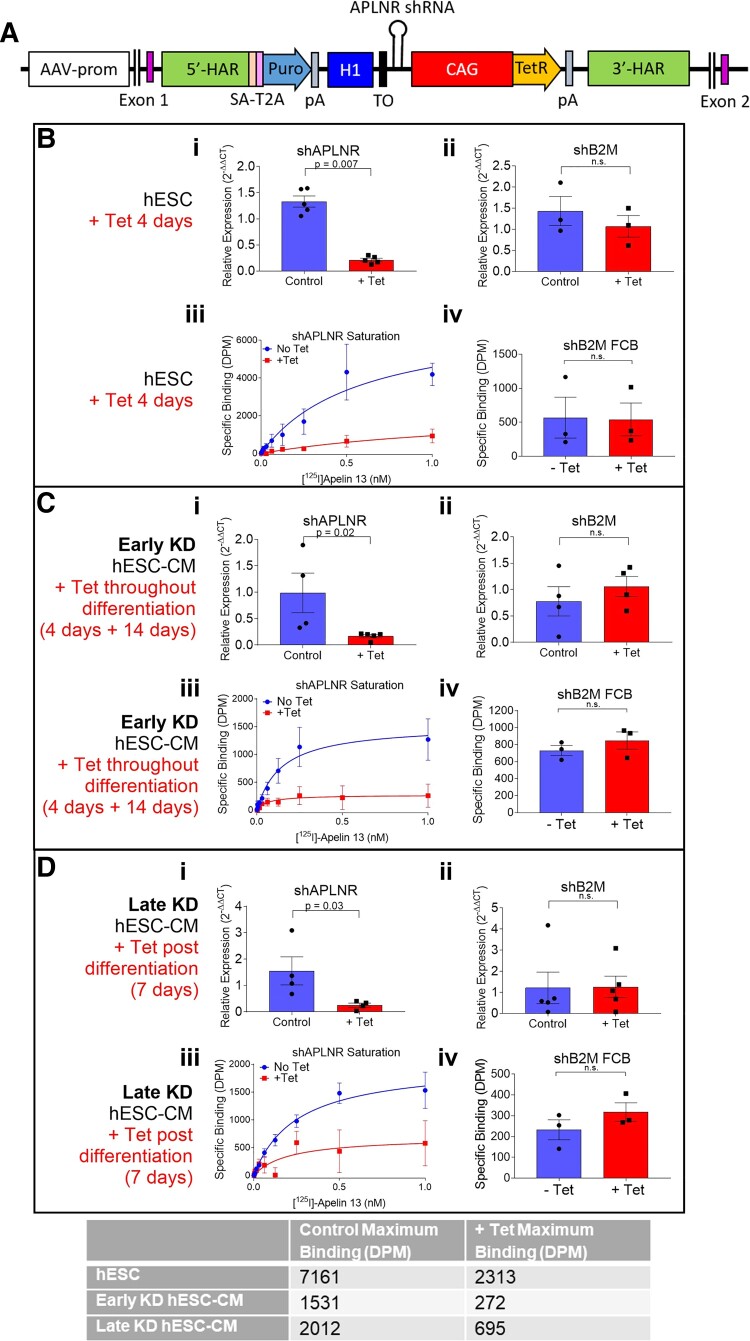
Generation and validation of a novel apelin receptor single step inducible knockdown system (shAPLNR). (*A*) Transgene generated containing *APLNR* shRNA targeted to the *AAVS1* locus. 5′-HAR/3′-HAR, upstream/downstream homology arm; SA, splice acceptor; T2A, self-cleaving T2A peptide; Puro, puromycin resistance; pA, polyadenylation signal; H1, H1 promoter; CAG, CAG promoter; TO, Tet operon; tetR, tetracycline-controlled repressor. (*B*) Expression of apelin receptor in hESCs cultured with or without tetracycline for 4 days. Comparison of relative expression (wrt shB2M + Tet) of apelin receptor gene (*APLNR*) for hESCs expressing shRNA directed against the (i) apelin receptor (*n* = 5, *P* < 0.001) or (ii) control line expressing shRNA directed against the *B2M* gene (*n* = 3). Expression levels compared by unpaired, two-tailed Student’s *t*-test. (iii) Saturation specific [^125^I]apelin-13 binding in hESCs expressing shAPLNR. (iv) Specific binding of fixed concentration of [^125^I]apelin-13 in hESCs expressing shB2M transgene. Specific binding levels compared by unpaired, two-tailed Student’s *t*-test. *n* = 3 for all. (*C*) Expression of apelin receptor in early knockdown hESC-CMs. Comparison of relative expression (wrt shB2M + Tet) of *APLNR* for hESC-CMs expressing shRNA directed against the (i) apelin receptor (control *n* = 4, +Tet *n* = 5, *P* = 0.04) or (ii) control line expressing shRNA directed against the *B2M* gene (*n* = 4). Expression levels compared by unpaired, two-tailed Student’s *t*-test. (iii) Saturation specific [^125^I]apelin-13 binding in hESC-CMs carrying the shAPLNR transgene. (iv) Specific binding of fixed concentration of [^125^I]apelin-13 in hESC-CMs expressing shB2M transgene. Specific binding levels compared by unpaired, two-tailed Student’s *t*-test. *n* = 3 for all. (*D*) Expression of apelin receptor in late knockdown hESC-CMs. Comparison of relative expression (wrt shB2M + Tet) of *APLNR* for hESC-CMs expressing shRNA directed against the (i) apelin receptor (*n* = 3, *P* = 0.01) or (ii) control line expressing shRNA directed against the *B2M* gene (*n* = 4). Expression levels compared by unpaired, two-tailed Student’s *t*-test. (iii) Saturation specific [^125^I]apelin-13 binding in hESC-CMs carrying the shAPLNR transgene. (iv) Specific binding of fixed concentration of [^125^I]apelin-13 in hESC-CMs expressing shB2M transgene. Specific binding levels compared by unpaired, two-tailed Student’s *t*-test. *n* = 3 for all. Inset table displays maximum binding in the three knockdown conditions for control and tetracycline treated cells. Data represent mean ± SEM.

*Knockdown of apelin receptor gene*: In undifferentiated hESCs expressing the transgene carrying shRNA targeting the apelin receptor (*Figure 2A*), expression of the *APLNR* gene following 4 days of tetracycline treatment was reduced by ∼85% (*Figure 2Bi*). In contrast, in control cells containing an identical transgene, except with the shRNA targeting the beta-2 microglobulin gene (shB2M), tetracycline inclusion had no effect on *APLNR* expression (*Figure 2Bii*). Next, shAPLNR hESCs were induced to differentiate to cardiomyocytes from hESCs cultured with or without tetracycline for 4 days prior, with tetracycline treatment maintained throughout differentiation (early knockdown). Expression of the apelin receptor gene was reduced by ∼90% with early knockdown, but the expression was unchanged in control shB2M cardiomyocytes (*Figure 2Ci and ii*). In late knockdown differentiated hESC-CMs cultured with tetracycline for 7 days post-completion of differentiation, *APLNR* expression was reduced by ∼85% but remained unchanged in shB2M cells (*Figure 2Di and ii*). Note that hESCs and hESC-CMs used to study the knockdown of the apelin receptor were distinct differentiations, carrying the shAPLNR transgene, vs. the wild-type differentiations used to determine expression of the apelin receptor and its endogenous ligands (observed in *Figure 1*).

*Knockdown of apelin receptor protein*: Following culture in the presence of tetracycline for 4 days, binding of [^125^I]apelin-13 was almost completely abolished in hESCs, indicating low expression of functional apelin receptor protein. Conversely, there was no difference in binding of a fixed concentration of [^125^I]apelin-13 between control and tetracycline treated shB2M hESCs (*Figure 2Biii and iv*). In early knockdown hESC-CMs, [^125^I]apelin-13 binding was greatly reduced, again indicating low expression of apelin receptor protein, with binding unaffected in hESC-CMs expressing the shB2M transgene (*Figure 2Ciii and iv*). The same was seen for late knockdown hESC-CMs, with [^125^I]apelin-13 binding much reduced in shAPLNR hESC-CMs but unchanged in shB2M cells (*Figure 2Diii and iv*). For all three knockdown conditions, [^125^I]apelin-13 binding affinity was unchanged with tetracycline treatment, but maximum-specific binding was much reduced (*Figure 2*, inset table). Furthermore, the binding of anti-apelin receptor antibody was much reduced in early apelin receptor knockdown hESC-CMs (see Supplementary material online, *Figure S2*).

### 3.3 Early apelin receptor knockdown decreases cardiomyocyte differentiation efficiency

**Figure 3:**
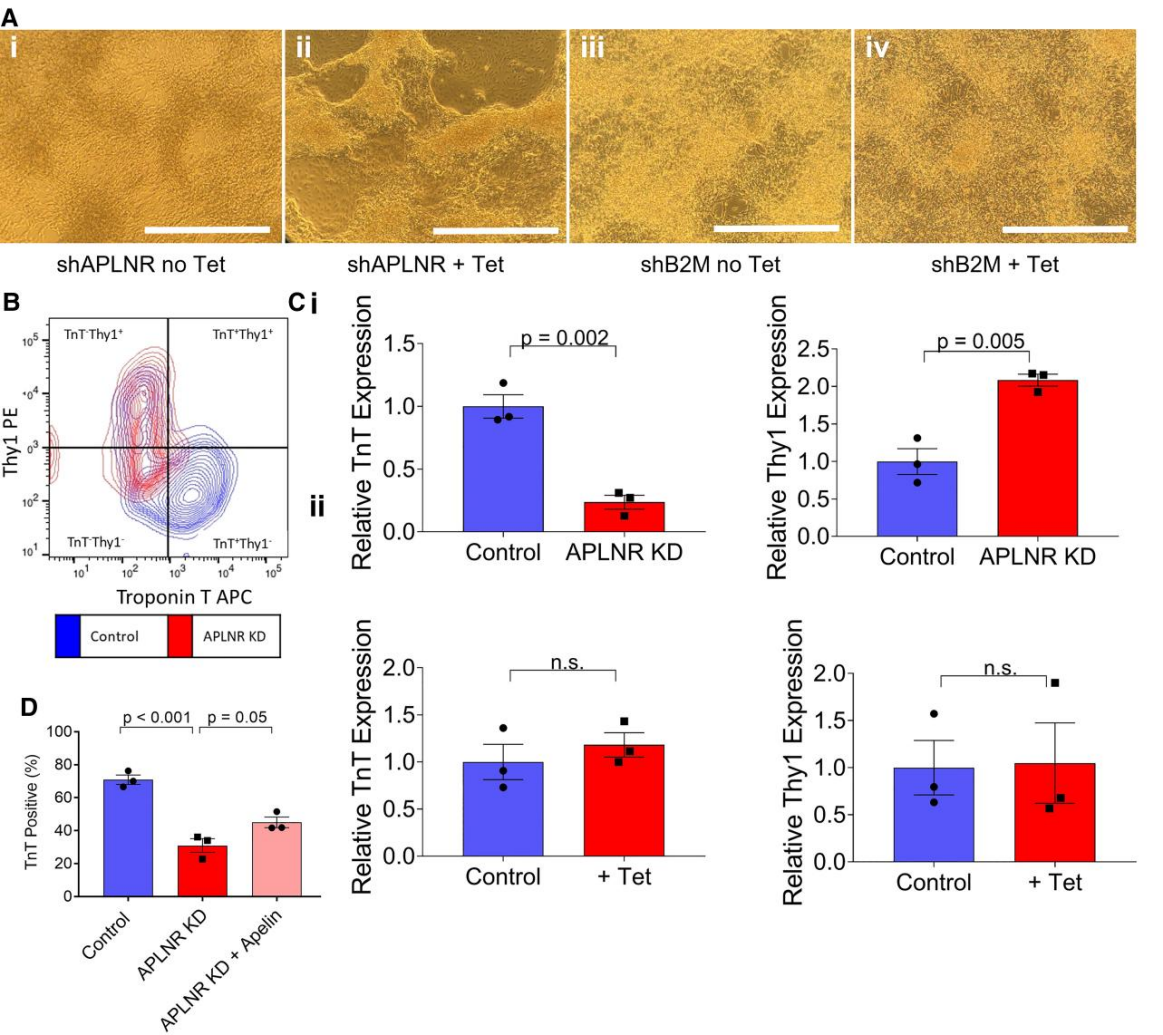
Early *APLNR* knockdown reduces differentiation efficiency of hESC-CMs and increases number of cells with fibroblast like identity. (*A*) Representative brightfield images of hESC-CMs carrying shAPLNR transgene cultured (i) without or (ii) with tetracycline (+Tet) throughout differentiation. (iii) and (iv) represent control cells carrying shB2M transgene cultured without or with tetracycline throughout differentiation. Scale bar = 200 µm. (*B*) Representative flow cytometry plot of control and *APLNR* knockdown hESC-CMs co-stained for cardiac troponin T (TnT, APC) and Thy1 (PE). (*C*) Quantification of TnT and Thy1 relative expression from flow cytomteric co-stain for (i) *APLNR* knockdown hESC-CMs relative to control (*P* = 0.002 for TnT, *P* = 0.005 for Thy1) and (ii) shB2M with tetracycline relative to control. *n* = 3 for all, compared by unpaired, two-tailed Student’s *t*-test. (*D*) TnT positive percentage from flow cytometric analysis of control, *APLNR* knockdown hESC-CMs and *APLNR* knockdown hESC-CMs cultured in the presence of 10 nM [Pyr^1^]apelin-13 throughout differentiation (+Apelin), *n* = 3. Means compared by one-way ANOVA followed by Tukey’s *post hoc* test. Data represent mean ± SEM.

We next examined the effects of early apelin receptor knockdown on hESC-CM differentiation. hESCs were treated with tetracycline for 4 days before initiating differentiation, and cells were then maintained in tetracycline throughout subsequent culture, to ensure continued *APLNR* knockdown. Distinct differences were observed between control cells and *APLNR* knockdown cells, with control lines forming homogenous sheets of beating cardiomyocytes, whilst in contrast the *APLNR* knockdown line grew as patches of clumped cardiomyocytes interspersed with fibroblasts (*Figure 3A*). *APLNR* knockdown also had marked consequences on observed spontaneous contraction. In control cells, strong and synchronous contraction was recorded (Supplementary material online, Video S1 and*Figure S3*). Conversely, when focusing on areas of cardiomyocytes in the knockdown line, the contraction was not in a homogenous synchronous beating pattern but random and weaker, with some differentiated cardiomyocytes displaying no contraction at all (Supplementary material online, Videos S2–S4).

To quantify the effect of *APLNR* knockdown on hESC-CM differentiation efficiency, cells were co-stained for the cardiac marker TnT and fibroblast marker Thy1 (*Figure 3B and C*). There was a significant increase in Thy1 positive cells in *APLNR* knockdown compared with wild-type control. In tandem, there was a decrease in TnT expression, indicating a decrease in cardiomyocyte differentiation efficiency and an increase in the number of cells acquiring a fibroblast identity with early *APLNR* knockdown. Cardiomyocyte differentiation efficiency was unaffected in shB2M control cells cultured in the presence of tetracycline (*Figure 3Cii*). In an attempt to rescue differentiation efficiency, [Pyr^1^]apelin-13 (10 nM) was included in the culture medium throughout the differentiation of shAPLNR hESCs alongside tetracycline (*Figure 3D*). With apelin inclusion, TnT positive percentage was increased by ∼15%, indicating some improvement of the *APLNR* knockdown phenotype.

To determine at which stage of differentiation the apelin receptor knockdown mediates its action on cardiomyocyte differentiation efficiency, qRT-PCR was performed at key stages throughout the differentiation protocol. The expression of standard stage-specific makers was quantified and compared between control and *APLNR* knockdown cells. Supplementary material online, *Figure S4* shows that for all intermediate stages and makers examined no significant difference was found, suggesting that the apelin receptor might be involved in the final stages of differentiation at the onset of contraction. This is consistent with the morphological changes seen in *APLNR* knockdown cells compared with control. As shown in the time course of images throughout differentiation (see Supplementary material online, *Figure S5*), the morphological features of *APLNR* knockdown and control cells appear comparable until the late stages of differentiation.

To further investigate the stage at which apelin receptor knockdown has its effect, knockdown was induced at key differentiation stages (mesoderm, cardiogenic mesoderm, cardiac progenitors) and TnT positive percentage was determined upon completion of differentiation (see Supplementary material online, *Figure S6*). Inducing *APLNR* knockdown at the mesoderm stage significantly reduced differentiation efficiency, with knockdown at subsequent stages having no significant effect on TnT positive percentage. Inducing apelin receptor knockdown at the different differentiation stages had no effect on Thy1 expression.

### 3.4 Early apelin receptor knockdown prolongs voltage sensing in hESC-CMs

**Figure 4:**
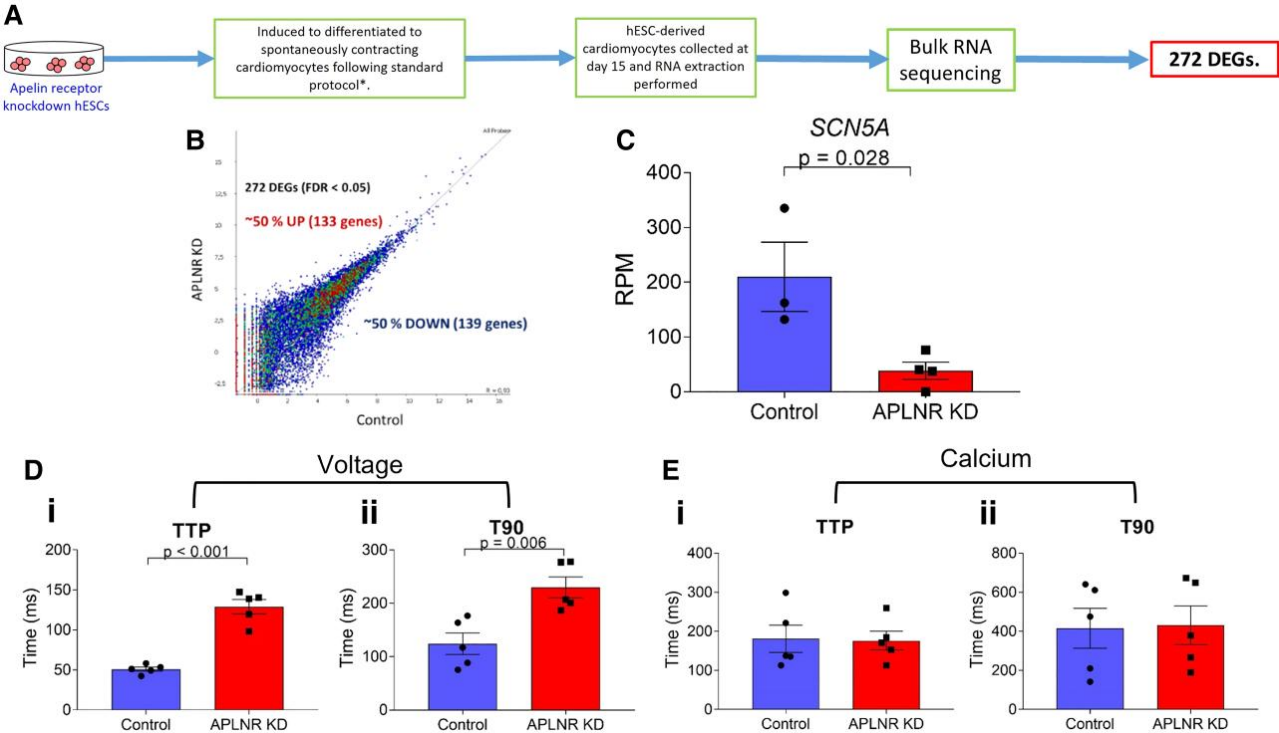
*APLNR* knockdown throughput differentiation (early knockdown) has functional consequences on hESC-CMs. (*A*) Workflow for bulk RNA-sequencing of *APLNR* knockdown hESC-CMs. (*B*) Plot representing number of up- and down-regulated differentially expressed genes (DEGs) in *APLNR* knockdown hESC-CMs compared with control. (*C*) Change in expression of *SCN5A* gene encoding the cardiac sodium channel Na_v_1.5 with *APLNR* knockdown. Control *n* = 3, *APLNR* knockdown *n* = 4, means compared by two-tailed Student’s *t*-test, *P* = 0.028. (*D*) (i) Time to peak (TTP) (*P* < 0.001) and (ii) time to 90% decay (T90) (*P* = 0.006) of voltage-sensitive dye in paced control and *APLNR* knockdown hESC-CMs. *n* = 5, means compared by unpaired, two-tailed Student’s *t*-test. (*E*) (i) TTP and (ii) T90 of calcium-sensitive dye in paced control and *APLNR* knockdown hESC-CMs. *n* = 5, means compared by unpaired, two-tailed Student’s *t*-test. Data represent mean ± SEM.

The functional consequences of early *APLNR* knockdown on hESC-CMs and the potential mechanistic basis for any changes seen were next investigated. RNA-sequencing was performed (*Figure 4A*) on early *APLNR* knockdown hESC-CMs and compared with control hESC-CMs. Our analysis identified 272 differentially expressed genes with a fold change of >log2(1.5) and a false discovery rate (FDR) of <0.05 with early *APLNR* knockdown, with 133 up-regulated and 139 down-regulated (*Figure 4B* and Supplementary material online, *Excel Files S1 and S2*). eXploring Genomic Relations (XGR) pathway analysis^31^ was performed, with results displayed in Supplementary material online, *Table S1*.

RNA-sequencing identified the cardiac sodium channel Na_V1.5_ gene (*SCN5A*) as the third most significantly down-regulated gene with *APLNR* knockdown (log2 fold change = −2.32, *Q*-value = 0.02) (*Figure 4C*). This was chosen for further characterization as the top druggable and physiologically relevant target, by comparison with the IUPHAR GuidetoPharmacology Database.^32^ The *SCN5A* gene encodes the α-subunit of the Na_V1.5_ voltage-dependent cardiac sodium channel, which is essential for depolarization and the initiation and conduction of the cardiac action potential.^33,34^ We, therefore, examined the effect of early *APLNR* knockdown on hESC-CM voltage sensing, using the voltage-sensitive FluoVolt Membrane Potential dye. Interestingly, *APLNR* knockdown was found to significantly increase both waveform time to peak and time to 90% decay (*Figure 4D*). Voltage signalling is intrinsically linked to cardiomyocyte contractility by the process known as excitation–contraction coupling, in which generation of an action potential and the associated ion fluxes results in muscle contraction.^35^ Therefore, we hypothesized that the prolonged voltage sensing seen in the *APLNR* knockdown cardiomyocytes may result in defective contractility.

Calcium is also essential for excitation–contraction coupling, with tight regulation of changes in intracellular calcium concentration induced by the cardiac action potential critical for cardiomyocyte contractility,^35^ therefore effect of early *APLNR* knockdown on intracellular calcium signalling was also examined. In contrast with what was observed for voltage sensing, time to peak and time to 90% decay of Fluo4-AM calcium-sensitive dye was unchanged in *APLNR* knockdown hESC-CMs compared with control (*Figure 4E*).

### 3.5 Apelin receptor knockdown in differentiated hESC-CMs has detrimental effects on contractility in 3D engineered heart tissues

**Figure 5:**
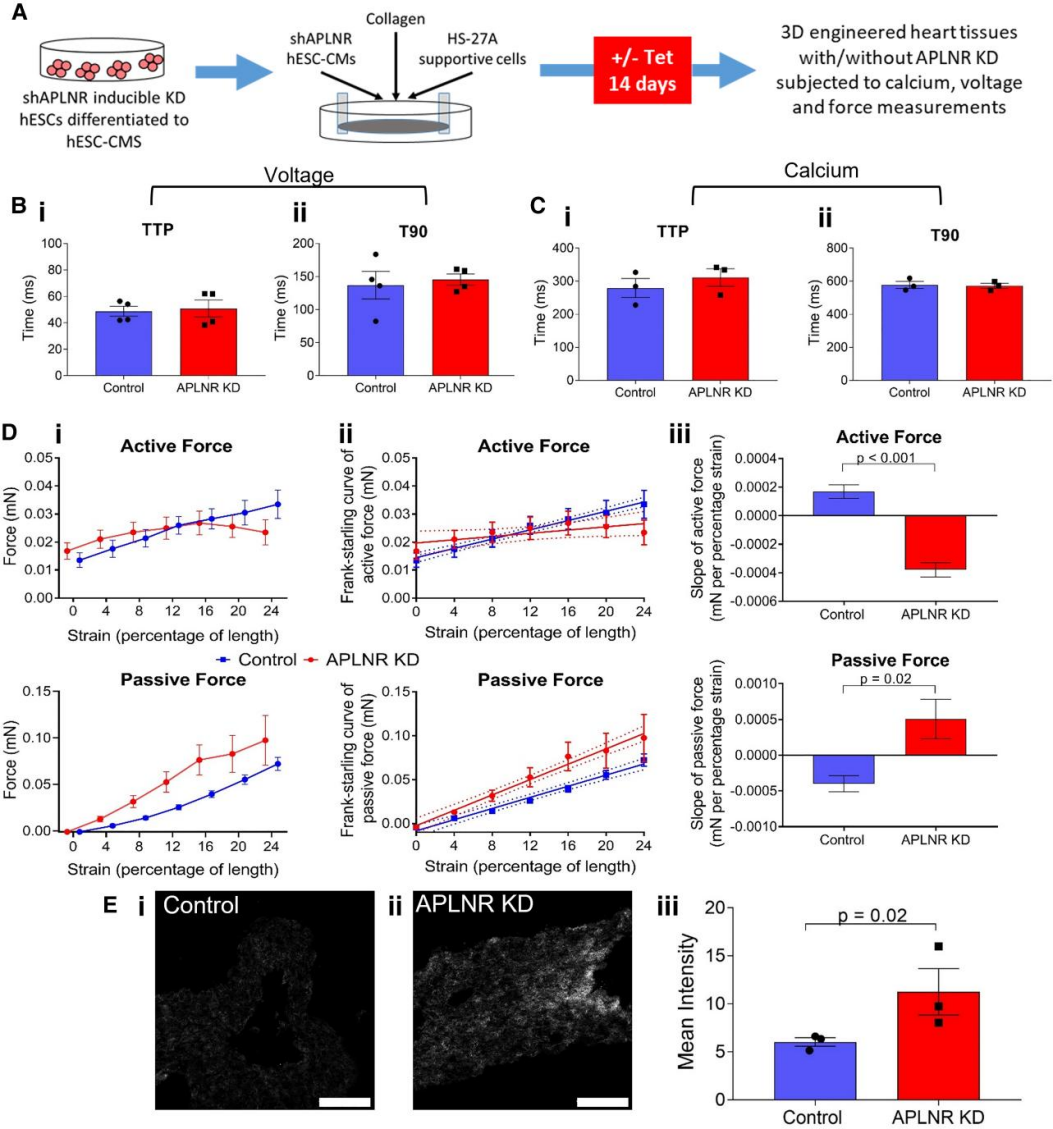
*APLNR* knockdown has detrimental effects on contractility in 3D engineered heart tissues (EHT). (*A*) Schematic of workflow used to produce *APLNR* knockdown EHTs. (*B*) (i) Time to peak (TTP) and (ii) time to 90% decay (T90) of voltage-sensitive dye in paced control and *APLNR* knockdown EHTs. *n* = 4, means compared by unpaired, two-tailed Student’s *t*-test. (*C*) (i) TTP and (ii) T90 of calcium-sensitive dye in paced control and *APLNR* knockdown hESC-CMs. *n* = 3, means compared by unpaired, two-tailed Student’s *t*-test. (*D*) (i) Active and passive force produced by control and *APLNR* knockdown EHTs as measured by force transducer in response to increasing strain. (ii) Linear regression of force produced to generate Frank-Starling curve of active and passive force. (iii) Slope of generated Frank-Starling curve of active and passive force. *n* = 4, means compared by unpaired, two-tailed Student’s *t*-test. For active force *P* < 0.001, for passive force *P* = 0.02. (*E*) Representative images of (i) control and (ii) *APLNR* knockdown EHTs imaged using second-harmonic imaging microscopy to visualize collagen. (iii) Quantification of mean pixel intensity of control and *APLNR* knockdown EHT collagen signal. *n* = 3, means compared by unpaired, two-tailed Student’s *t*-test, *P* = 0.02. Data represent mean ± SEM.

Having established the importance of the apelin receptor throughout cardiomyocyte differentiation, we next investigated the effects of apelin receptor knockdown in differentiated hESC-CMs. 3D EHTs were generated and *APLNR* knockdown induced (*Figure 5A*), which was confirmed by qRT-PCR (see Supplementary material online, *Figure S7*).

Control and *APLNR* knockdown EHTs were loaded with either voltage or calcium-sensitive dye (*Figure 5B and C*, respectively) and time to peak and time to 90% decay were determined. Interestingly, in contrast to results seen with early knockdown, there was no effect of *APLNR* knockdown on voltage sensing. Consistent with early knockdown results and previously published studies, calcium sensing was also unaffected.

To measure contractility, EHTs were subjected to stretch and electrical pacing (*Figure 5D*), with control EHTs following the Frank-Starling mechanism as shown previously,^28,29^ generating increasing active force in response to increased strain. In contrast, *APLNR* knockdown EHTs were less capable of responding to stretch, with the slope of active force significantly reduced compared with control. Related to this, *APLNR* knockdown in EHTs resulted in a higher passive force compared with control EHTs, indicating decreased compliance and increased tissue stiffness. Consistent with the increased stiffness, *APLNR* knockdown EHTs expressed increased collagen levels compared with control (*Figure 5E* and Supplementary material online, *Figure S8*), suggesting loss of the apelin receptor is pro-fibrotic. Owing to the poor differentiation efficiency, early apelin receptor knockdown resulted in insufficient cardiomyocytes to be able to generate viable EHTs to carry out similar measurements.

## 4. Discussion

We have examined, for the first time, the role of the apelin receptor in hESC-CM differentiation and function. Although the left ventricle samples will not be a pure cardiomyocyte population, hESC-derived cardiomyocytes express the apelin receptor at a similar level to that seen in the adult heart, suggesting this system is a suitable model to investigate the role of the apelin receptor in cardiomyocyte function. Significantly, we have also shown that hESCs express apelin receptor protein that is capable of binding apelin ligand.

We have generated an apelin receptor tetracycline-dependent inducible knockdown system, demonstrating significant apelin receptor knockdown at the gene and protein level in hESCs, hESC-CMs treated with tetracycline throughout differentiation (early knockdown), and in hESC-CMs treated with tetracycline upon completion of differentiation (late knockdown). To our knowledge, not only is this the first use of the sOPTiKD system to knockdown the apelin receptor, but also the first application of this system to knockdown a GPCR in hESCs or hESC-CMs. This is of particular note as GPCRs are challenging to knockdown due to their low expression level.

By inducing knockdown throughout differentiation, these data demonstrate the importance of the apelin receptor in cardiomyocyte differentiation and confirm its identity as a key signalling system for cardiomyocyte development. The results are consistent with previous studies in two animal models (mouse and zebrafish) but in clinically relevant human cardiomyocytes.^1,5^

A previously published study demonstrated inclusion of apelin in culture medium increased cardiac differentiation efficiency of hESCs to contractile embryoid bodies.^36^ Furthermore, embryoid body cardiomyocyte differentiation of apelin receptor knockout mouse ESCs was impaired.^37^ In agreement with this, apelin receptor knockdown reduced the number of cells staining positive for the cardiomyocyte marker TnT, whilst inclusion of apelin peptide in the culture medium throughout differentiation increased the efficiency of cardiomyocyte differentiation. Importantly, the differentiation protocol used here is better defined, generating a high percentage of hESC-CMs reproducibly.^20,38^ Interestingly, apelin receptor knockdown at the mesoderm stage also significantly reduced hESC-CM differentiation efficiency, whilst knockdown at later stages had no effect. This suggests that the apelin receptor may be acting in the early stages of differentiation, although its effects are not phenotypically visible until the later stages of differentiation.

Early apelin receptor knockdown also had functional consequences, with hESC-CMs displaying prolonged voltage sensing and decreased expression of the gene encoding the Na_V1.5_ cardiac sodium channel. Previous studies have suggested the involvement of the apelin signalling system in cardiac electrophysiology, with plasma apelin levels found to be reduced in atrial fibrillation patients, which can be restored by long-term cardiac resynchronization therapy.^39,40^ Additionally, apelin peptide has previously been shown to modulate sodium currents in cardiomyocytes, increasing action potential conduction velocity.^41,42^ The prolongation of voltage signalling in apelin receptor knockdown hESC-CMs suggests dysregulation. We hypothesized that excitation–contraction coupling may be disrupted, leading to defects in contractility. In excitation–contraction coupling, calcium signalling is essential, with intracellular calcium increase following the depolarization induced by the cardiac sodium channels.^43^ Crucially, however, apelin receptor knockdown had no effect on intracellular calcium signalling. Our results support previous proposals that activation of the apelin signalling pathway can modulate voltage signalling to increase intracellular pH, resulting in increased myofilament sensitivity to calcium, without increasing calcium concentration, contributing to the inotropic effect of apelin receptor activation.^42^ Consistent with this, no difference was seen in calcium transients in isolated cardiomyocytes from apelin receptor knockout mice compared with wild-type controls.^2^

Finally, late apelin receptor knockdown in 3D EHTs was found to have detrimental effects on contractile properties, reducing contractility and increasing stiffness compared with control, similar to genetic disruption of *apln* in mice.^2^ Our results support the role of apelin signalling in heart contractility, and the link between reduced apelin signalling and the decreased contractile performance observed in heart failure patients.^44^

The increased stiffness of *APLNR* knockdown EHTs was associated with increased collagen deposition. Apelin has previously been shown to have anti-fibrotic effects, with the administration of apelin in wild-type mice reducing angiotensin II (AngII) induced cardiovascular fibrosis via regulation of PAI-1 gene expression and increased production of nitric oxide.^45^ Furthermore, in rats, apelin treatment was found to reduce fibrosis following myocardial infarction, by inhibiting AngII-induced NF-κB activation.^46^ Additionally, apelin significantly improved ventricular remodelling and function, as well as attenuating established hypertrophy and fibrosis, when administered 14 days after surgically induced pressure overload.^47^ Our results showing the effects of loss of apelin receptor signalling in a human system support the anti-fibrotic role of the apelin receptor.

Notably, the apelin receptor knockdown EHTs recapitulate the phenotype of what is seen in a failing cardiomyocyte, with previous studies reporting significant increases in passive force in isolated cardiomyocytes of diastolic heart failure patients,^48^ and preparations from both right and left ventricles of failing hearts producing significantly reduced active force.^49^ Associated with this, the increased collagen deposition in *APLNR* knockdown EHTs correlates with the increase in fibrosis seen in the failing human heart.^50^ Furthermore, in EHTs produced from induced pluripotent stem cell-derived CMs (iPSC-CMs) carrying a mutation associated with inherited dilated cardiomyopathy, the amplitude of twitch force was much reduced compared with EHTs produced from healthy iPSC-CMs.^51^

These data confirm that hESCs and hESC-CMs express functional apelin receptor protein at the plasma membrane at similar levels to that seen in adult cardiomyocytes. Using genetic manipulation, we have identified a key role for the apelin receptor in hESC-CM growth factor-driven differentiation and function. The results suggest this system may be used for further investigations into the role of the apelin receptor in cardiovascular disease and provides a platform with the potential for high throughput screening of novel therapeutic agents.

## Supplementary material

Supplementary material is available at *Cardiovascular Research* online.

## Authors’ contributions

R.G.C.M., M.T.C, T.L.W., S.B., R.E.K., and A.L.P. designed and/or carried out experiments and/or data analysis. W.G.B. contributed expertise in genetic editing. E.L.R. and E.E.D. contributed expertise in RNA-sequencing and analysis. J.J.M., S.S., and A.P.D. designed and supervised experiments, performed data analysis, contributed grant support and facilities. All authors contributed to the writing and/or review of the manuscript.

## Supplementary Material

cvac065_Supplementary_Data

## Data Availability

The data that support the findings of this study are available from the corresponding author upon reasonable request.
