# Supplementary material for: Inducible apelin receptor knockdown reduces differentiation efficiency and contractility of hESC-derived cardiomyocytes
Source: Cardiovasc Res. 2022 May 16;119(2):587–98. doi: 10.1093/cvr/cvac065 (PMC10064845; doi:10.1093/cvr/cvac065)
Supplement: cvac065_Supplementary_Data [file cvac065_supplementary_data.zip › RM0703CVRESRevisions_Supplementary Material_Blue.docx]

**Supplementary Materials**

**Inducible apelin receptor knockdown reduces differentiation efficiency and contractility of hESC-derived cardiomyocytes**

Robyn G.C. Macrae^1,2^, Maria T. Colzani^2^, Thomas L. Williams^1^, Semih Bayraktar^2^, Rhoda E. Kuc^1^, Anna L. Pullinger^1,2^, William G. Bernard^2^, Emma L. Robinson^3^, Emma E. Davenport^4^, Janet J. Maguire^1^, Sanjay Sinha^2§^, Anthony P. Davenport^1§^*.

1. Experimental Medicine and Immunotherapeutics, University of Cambridge, Addenbrooke’s Hospital, Cambridge, U.K.

2. Wellcome-MRC Cambridge Stem Cell Institute, Jeffrey Cheah Biomedical Centre, University of Cambridge, Cambridge, U.K.

3. School of Medicine, Division of Cardiology, University of Colorado Denver, Aurora, USA.

4. Wellcome Sanger Institute, Cambridge, UK.

^§^Joint senior authors

**Supplementary Tables**

| \| **Term Name** \| **Z-score** \| **P-value** \| **FDR** \| **Number of genes overlapped** \| **Genes** \| \| --- \| --- \| --- \| --- \| --- \| --- \| \| **Signaling events mediated by focal adhesion kinase** \| 5.36 \| 0.000029 \| 0.00069 \| 6 \| ARHGAP26, BCAR1, FYN, NCK2, PTPN21, VCL \| \| **E-cadherin signaling in keratinocytes** \| 4.7 \| 0.00027 \| 0.0032 \| 3 \| FMN1, FYN, JUP \| \| **Ephrin B reverse signaling** \| 3.73 \| 0.0011 \| 0.0083 \| 3 \| FGA, FYN, NCK2 \| \| **C-MYB transcription factor network** \| 3.27 \| 0.0018 \| 0.0083 \| 5 \| CREBBP, NCOR1, PAX5, SP1, TAB2 \| \| **Alpha4 beta1 integrin signaling events** \| 3.49 \| 0.0016 \| 0.0083 \| 3 \| BCAR1, PRKACB, THBS1 \| \| **p53 pathway** \| 3.25 \| 0.0021 \| 0.0083 \| 4 \| ATM, CHEK1, CREBBP, FBXO11 \| \| **ErbB4 signaling events** \| 3.15 \| 0.0027 \| 0.0092 \| 3 \| FYN, NCOR1, TAB2 \| \| **Signaling events mediated by VEGFR1 and VEGFR2** \| 2.85 \| 0.0041 \| 0.01 \| 4 \| FYN, NCK2, PTPRJ, VCL \| \| **Beta3 integrin cell surface interactions** \| 2.87 \| 0.0042 \| 0.01 \| 3 \| FGA, THBS1, TNC \| \| **Urokinase-type plasminogen activator (uPA) and uPAR-mediated signaling** \| 2.92 \| 0.0039 \| 0.01 \| 3 \| BCAR1, FGA, NCL \| \| **Posttranslational regulation of adherens junction stability and dissassembly** \| 2.63 \| 0.0063 \| 0.014 \| 3 \| CREBBP, FYN, JUP \| \| **Signaling events mediated by PTP1B** \| 2.46 \| 0.0084 \| 0.017 \| 3 \| BCAR1, FYN, YBX1 \| \| **PDGFR-beta signaling pathway** \| 2.15 \| 0.015 \| 0.027 \| 5 \| BCAR1, EPS8, FYN, NCK2, PTPRJ \| \| **Beta1 integrin cell surface interactions** \| 1.96 \| 0.019 \| 0.033 \| 3 \| FGA, THBS1, TNC \| \| **Regulation of Telomerase** \| 1.9 \| 0.021 \| 0.034 \| 3 \| ATM, NCL, SP1 \| \| **E2F transcription factor network** \| 1.74 \| 0.028 \| 0.042 \| 3 \| ATM, CREBBP, SP1 \| \| **Genes encoding structural ECM glycoproteins** \| 1.73 \| 0.032 \| 0.044 \| 6 \| EMILIN2, EYS, FGA, THBS1, TNC, TSPEAR \| \| **Regulation of nuclear SMAD2/3 signaling** \| 1.54 \| 0.038 \| 0.044 \| 3 \| CREBBP, NCOR1, SP1 \| \| **p73 transcription factor network** \| 1.61 \| 0.034 \| 0.044 \| 3 \| CHEK1, PRKACB, SP1 \| \| **Signaling events mediated by Hepatocyte Growth Factor Receptor (c-Met)** \| 1.59 \| 0.036 \| 0.044 \| 3 \| BCAR1, NCK2, PTPRJ \| \| **Integrin Signaling Pathway** \| 1.54 \| 0.038 \| 0.044 \| 3 \| ARHGAP26, BCAR1, FYN \| |
| --- | --- | --- | --- | --- | --- | --- | --- | --- | --- | --- | --- | --- | --- | --- | --- | --- | --- | --- | --- | --- | --- | --- | --- | --- | --- | --- | --- | --- | --- | --- | --- | --- | --- | --- | --- | --- | --- | --- | --- | --- | --- | --- | --- | --- | --- | --- | --- | --- | --- | --- | --- | --- | --- | --- | --- | --- | --- | --- | --- | --- | --- | --- | --- | --- | --- | --- | --- | --- | --- | --- | --- | --- | --- | --- | --- | --- | --- | --- | --- | --- | --- | --- | --- | --- | --- | --- | --- | --- | --- | --- | --- | --- | --- | --- | --- | --- | --- | --- | --- | --- | --- | --- | --- | --- | --- | --- | --- | --- | --- | --- | --- | --- | --- | --- | --- | --- | --- | --- | --- | --- | --- | --- | --- | --- | --- | --- | --- | --- | --- | --- | --- | --- |

**Supplementary Table 1**: Pathway analysis using XGR^31^ (<http://galahad.well.ox.ac.uk/>) showing pathways with enrichments, with FDR<0.05, listed by significance value.

**Supplementary Figures**


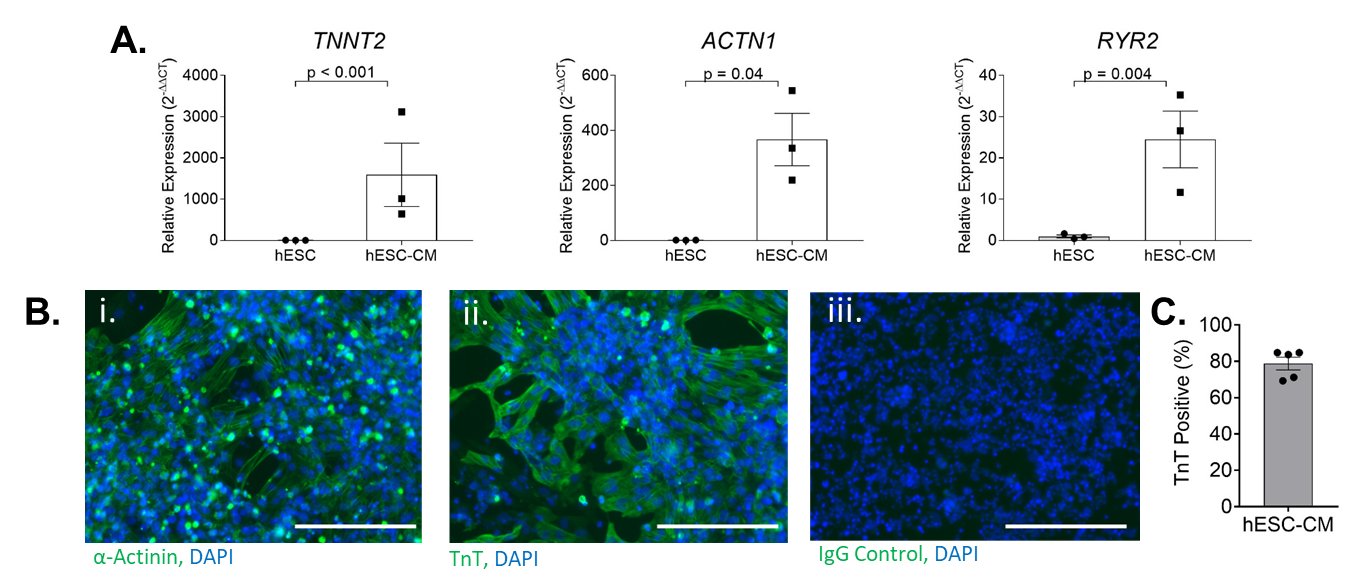


**Supplementary Figure 1**: hESC-CMs express standard cardiac markers at the gene and protein level. (**A**) Comparison of relative expression of genes encoding standard cardiac markers in hESCs and hESC-CMs by qRT-PCR. Expression displayed relative to mean expression in hESCs. n = 3, compared by unpaired, two-tailed Student’s t-test. For *TNNT2* p < 0.001, for *ACTN1* p = 0.04, for *RYR2* p = 0.004. (**B**) Representative images of hESC-CMs stained with antibody directed against (i) α-actinin (green), (ii) cardiac troponin T (TnT) (green) or (iii) isotype negative control. Blue = DAPI nuclear stain, scale bar = 200 μm. (**C**) Troponin T (TnT) positive percentage of hESC-CMs by flow cytometry, n = 5. Data represent mean±sem.


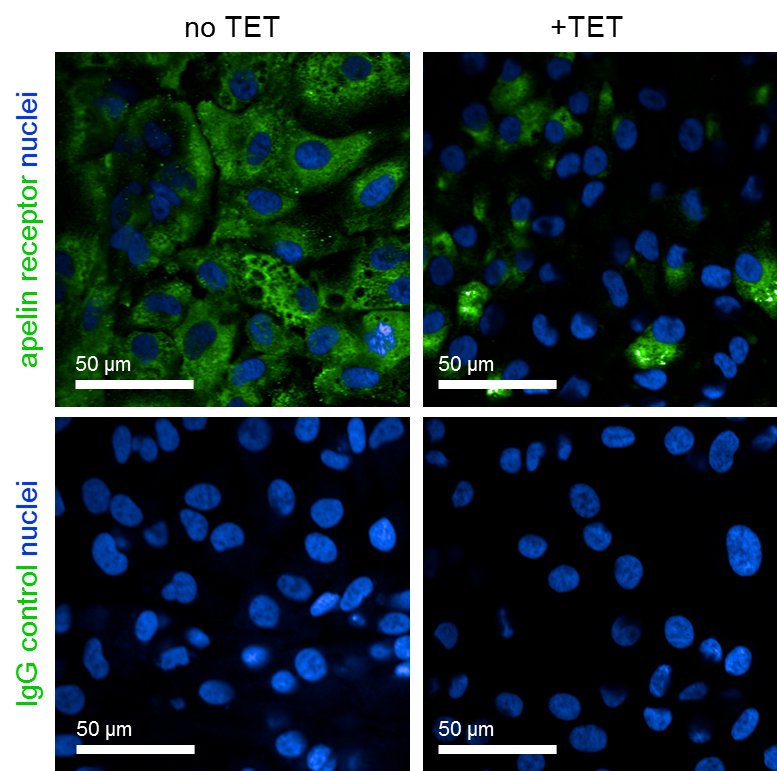


**Supplementary Figure 2**: Validation of apelin receptor antibody using *APLNR* knockdown hESC-CMS. Representative fluorescent confocal images of expression of apelin receptor using an apelin receptor antibody in hESC-CMs cultured in the absence (no TET, upper left) or presence of tetracycline (+ TET, upper right). Control hESC-CMs were treated with an IgG isotype control in the absence (no TET, bottom left) or presence of tetracycline (+ TET, bottom right). The data confirm specificity of the antibody and show that the receptor is expressed at lower levels in the presence of tetracycline.


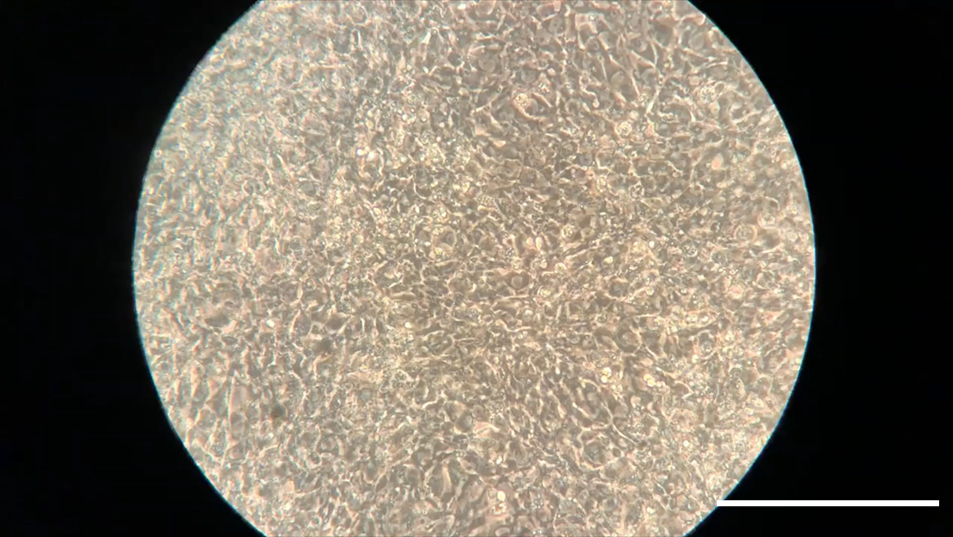


**Supplementary Figure 3**: Still from representative video of control hESC-CMs with scale bar for reference. Scale bar = 50 µm.


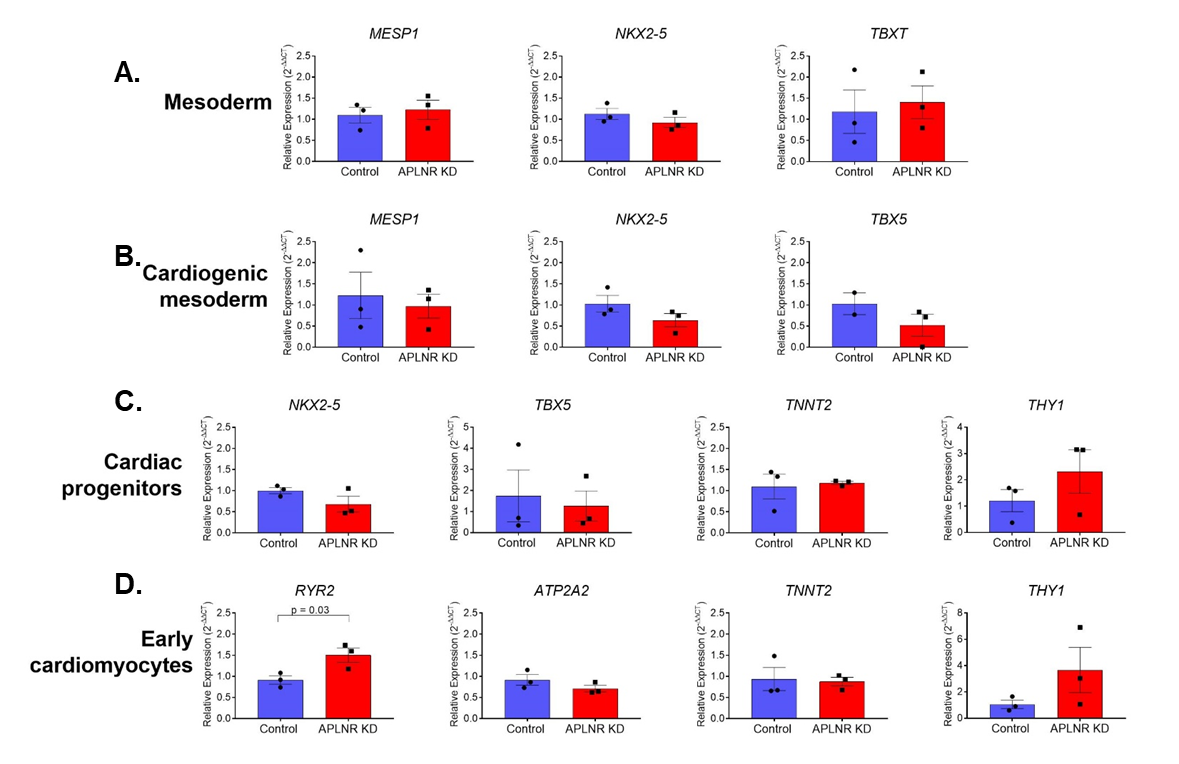


**Supplementary Figure 4**: Comparison of relative expression of stage specific markers across four stages of hESC-CM differentiation, (**A**) mesoderm, (**B**) cardiogenic mesoderm, (**C**) cardiac progenitors and (**D**) early cardiomyocytes for *APLNR* knockdown hESC-CMs compared to control. Expression displayed relative to mean expression in control for each gene. n = 3, compared by unpaired, two-tailed Student’s t-test. For early cardiomyocytes *RYR2* p = 0.03. Data represent mean±sem.


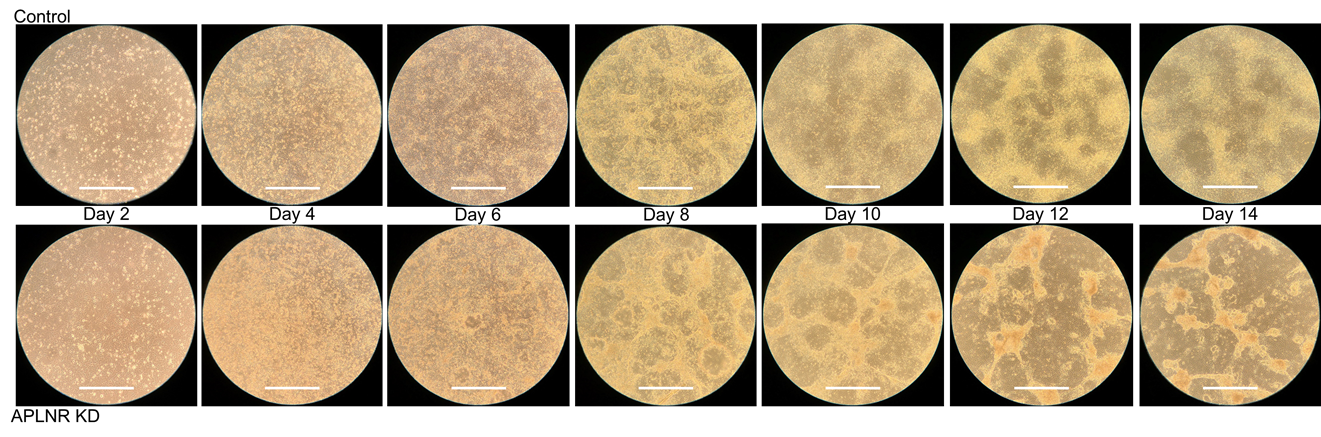


**Supplementary Figure 5**: Representative images of control (top) and *APLNR* knockdown (bottom) hESC-CMs throughout differentiation. Scale bar = 200 µm.


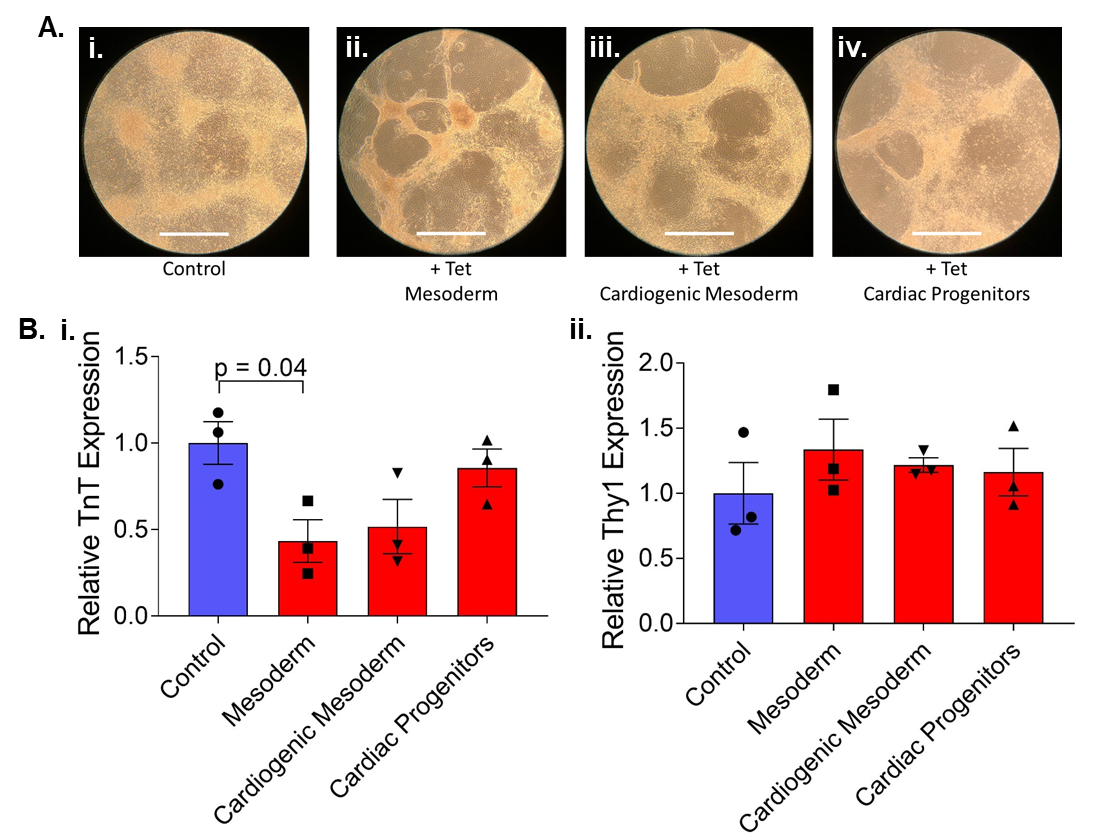


**Supplementary Figure 6**: (A) Representative images of hESC-CMs upon completion of differentiation with *APLNR* knockdown induced at key stages of differentiation. Scale bar = 200 µm. (B) Relative expression of (i) cardiac marker troponin T (TnT) and (ii) fibroblast marker Thy1 upon completion of differentiation in control hESC-CMs and hESC-CMs with apelin receptor knockdown induced at key differentiation stages (mesoderm, cardiogenic mesoderm and cardiac progenitors). Expression displayed relative to control expression. n=3 for all. Data compared by one way ANOVA with Tukey’s post hoc test. For mesoderm relative TnT expression p = 0.04. Data represent mean±sem.


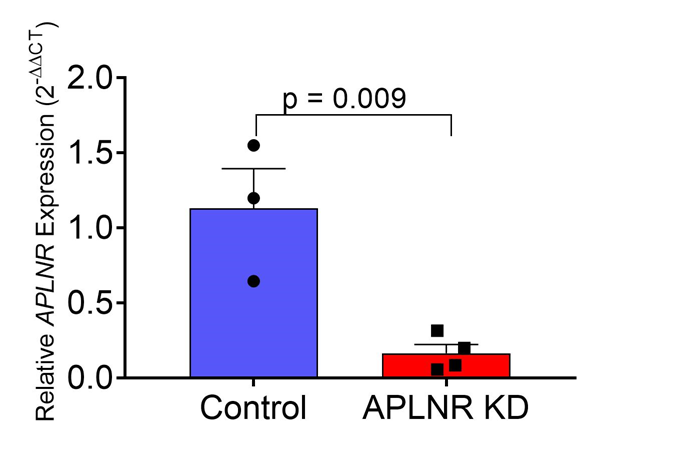


**Supplementary Figure 7**: Comparison of relative expression of the apelin receptor gene (*APLNR*) in control and *APLNR* knockdown EHTs after 14 days of tetracycline treatment. Control n = 3, apelin receptor knockdown n = 4, expression displayed relative to control, compared by unpaired two-tailed Student’s t-test, p = 0.009. Data represent mean±sem.


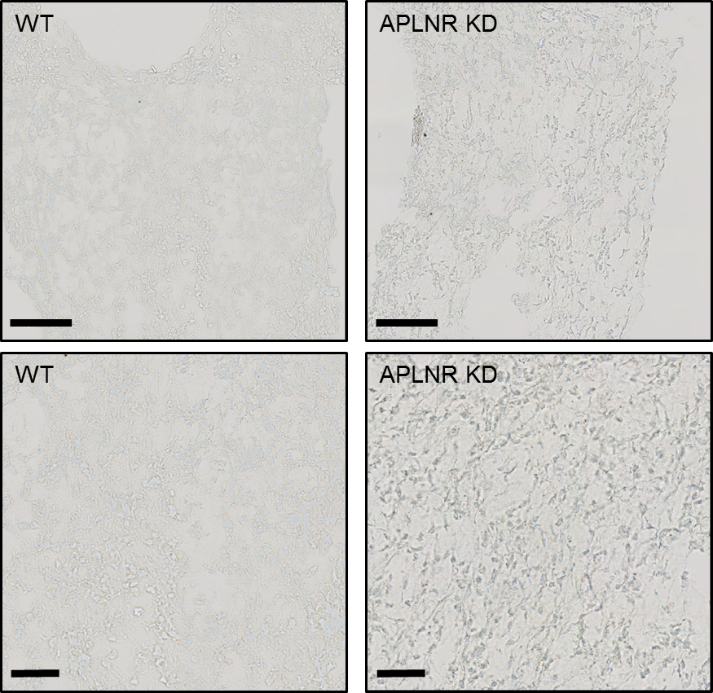


**Supplementary Figure 8**: Representative bright-field images of wild-type (WT, left) and apelin receptor knockdown (APLNR KD, right) EHTs treated with aniline blue collagen fibre stain. n = 3 stained in quadruplicate. Scale bars as indicated in figure.

**Legends for Video Files**

Video S1: Representative video of spontaneous contraction of control (no tetracycline) hESC-CMs.

Video S2: Representative video of spontaneous contraction of early *APLNR* KD (+ tetracycline throughout differentiation) hESC-CMs.

Video S3: Representative video of spontaneous contraction of early *APLNR* KD (+ tetracycline throughout differentiation) hESC-CMs.

Video S4: Representative video of spontaneous contraction of early *APLNR* KD (+ tetracycline throughout differentiation) hESC-CMs.

**Supplementary Methods**

**Cell culture**

hESC culture and hESC-CM differentiation

Pluripotent H9 hESCs (WiCell) were maintained in culture as described previously^18^. hESCs were grown as colonies in complete defined medium (CDM-BSA, Table S2), supplemented with Fibroblast Growth Factor 2 (FGF2, 25 ng/ml, Qkine Ltd) and Activin-A (10 ng/ml, Qkine Ltd), on culture plates previously coated with gelatin and incubated with MEF media overnight. Cells were passaged as necessary using collagenase IV (ThermoFisher).

A previously optimised protocol was used to direct differentiation to hESC-CMs (Figure 1A) (adapted from^19^). Briefly, hESCs were washed with PBS (Gibco) and detached by incubation with TryPLE (Gibco) (3 minutes at 37°C). Cells were counted using a Countess Automated Cell Counter (Invitrogen), with cells diluted 1:1 with 0.4% Trypan Blue solution (Gibco). hESCs were plated at a density of 8 x 10^5^ cells/well on Matrigel (Corning) coated plates. Following attachment, differentiation to mesoderm was induced by culturing in CDM-BSA media supplemented with FGF2 (20 ng/ml), Activin-A (50 ng/ml) and Bone Morphogenetic Protein 4 (BMP4, 10 ng/ml, R&D Systems) and also containing phosphoinositide 3-kinase inhibitor Ly294002 (10 µM, Stratech). Cells were incubated for 42 h at 37°C before changing media to CDM-BSA supplemented with FGF2 (8 ng/ml), BMP4 (10 ng/ml), retinoic acid (1 µM, Sigma Aldrich) and the WNT signalling pathway inhibitor IWR1-endo (1 ng/ml, Tocris). This was refreshed after 48 h, and following another 48 h replaced with CDM-BSA supplemented with FGF2 (8 ng/ml) and BMP4 (10 ng/ml). 48 h later, media was changed to unsupplemented CDM-BSA. CDM-BSA (with refresh every other day) was used to maintain hESC-CMs until robust beating was established. Independent replicates were considered to be hESC-CMs generated from distinct differentiations.

**Supplementary Table 2:** Primer sequences or IDs used for qRT-PCR.

| **Medium** | **Components** | **Concentration** | **Supplier** |
| --- | --- | --- | --- |
| CDM-BSA | IMDM:F12 (1:1) | - | ThermoFisher |
|  | Transferrin | 15 μg/ml | R&D Systems |
|  | Insulin | 7 μg/ml | Sigma Aldrich |
|  | Monothioglycerol | 450 mM | Sigma Aldrich |
|  | Chemically defined concentrated lipids | 1% | ThermoFisher |
|  | Bovine Serum Albumin | 5 mg/ml | Europa Bio Products |
|  | Penicillin-Streptomycin | 100 U/ml | ThermoFisher |
| MEF Medium | Advanced DMEM/F12 | - | ThermoFisher |
|  | Foetal Bovine Serum (FBS) | 10% | ThermoFisher |
|  | L-Glutamine | 1% | ThermoFisher |
|  | β-mercaptoethanol | 100 μM | Sigma Aldrich |
|  | Penicillin-Streptomycin | 100 U/ml | ThermoFisher |
| Gelatin | Embryo transfer water | - | Sigma Aldrich |
|  | Gelatin from porcine skin | 0.1% | Sigma Aldrich |
| Collagenase | Advanced DMEM/F12 | - | ThermoFisher |
|  | Knockout serum replacer (KSR) | 20% | ThermoFisher |
|  | L-Glutamine | 1% | ThermoFisher |
|  | Collagenase IV | 1 mg/ml | ThermoFisher |

HS-27A

HS-27A cells (ATCC) were maintained on gelatin coated plates in MEF media. Upon reaching confluence, cells were detached by incubating with TryPLE for 3 minutes at 37°C. Trypsin was neutralised, cells collected and pelted by centrifugation (300 xg, 3 minutes). Pellets were resuspended and plated in pre-prepared 0.1% gelatin coated plates.

**Gene expression analysis using quantitative real time polymerase chain reaction (qRT-PCR)**

RNA extraction was performed using the GenElute Total RNA Purification Kit (Sigma Aldrich) according to manufacturer’s protocol. Samples were eluted in 30 μl Nuclease Free Water (QIAGEN), with RNA concentration determined using a NanoDrop One (ThermoFisher). cDNA was produced from 1 μg of RNA using the Promega Reverse Transcription System.

qRT-PCR was performed for 45 cycles using the ABI 7500 Real-Time PCR System (Applied Biosystems) to analyse mRNA expression using 96-Well TaqMan Gene Expression Assays (Applied Biosystems) or 96-Well or 384-Well SYBR Green Based Assays (ThermoFisher) with primer sequences shown in Table S3. Human *18S* rRNA or *GAPDH* were used as house-keeping genes owing to their stable expression level across cells used. Relative expression was normalised to housekeeping gene expression using the 2(^-ΔΔCT^) method^22^.

| **Target** | **Assay Type** | **Primer Sequence or ID** | **Acquired From** |
| --- | --- | --- | --- |
| *18S* | TaqMan | Hs99999901_s1 | ThermoFisher |
| *APLNR* | TaqMan | Hs00270873_s1 | ThermoFisher |
| *APLN* | TaqMan | Hs00175572_m1 | ThermoFisher |
| *APELA* | TaqMan | Sense: GAAGAAGAAGAGGAGTGAAGGA  Antisense: CCATTCCAGGTGCTTTCAAAT | Primer Design |
| *GAPDH* | SYBR Green | FOR: AACAGCCTCAAGATCATCAGC  REV: GGATGATGTTCTGGAGAGCC | Sigma Aldrich |
| *TNNT2* | SYBR Green | FOR: CCCAATGGAGGAGTCCAAAC  REV: CCCGACGTCTCTCGATCC | Sigma Aldrich |
| *ACTN1* | SYBR Green | FOR: TCAACCACTTTGACCGGGAT  REV: GCAAATTCTGCTTCTCCCTGG | Sigma Aldrich |
| *RYR2* | SYBR Green | FOR: ACAACAGAAGCTATGCTTGGC  REV: GAGGAGTGTTCGATGACCACC | Sigma Aldrich |

**Supplementary Table 3:** Primer sequences or IDs used for qRT-PCR.

**Determination of protein content and saturation radioligand binding**

Pelleted hESC-CMs were resuspended in 1 ml ice-cold Tris-HCl (50 mM (Sigma Aldrich), pH 7.4) and triturated to induce hypotonic lysis. Samples were centrifuged (20,000 xg, 20 minutes at 4°C) and resuspended in ice-cold Tris-HCl buffer. Cell lysate was combined 1:2 with solubilisation buffer (0.5 M NaOH, 1% SDS) and incubated at 80°C for 30 minutes. Protein content was assessed using the DC Protein Assay (Bio-Rad) following the manufacturer’s protocol, using assay kit standards as a reference and solubilisation buffer alone as a blank. Absorbance was measured at 450 nm using FLUOstar® Omega Microplate Reader (BMG Labtech).

Saturation binding experiments were carried out in human ESC-derived CMs as described previously^25^ using [Glp^65^,Nle^75^,Tyr^77^][^125^I]apelin-13 (Perkin Elmer) (referred to as [^125^I]apelin-13 subsequently). All plasticware used was coated with Sigmacote siliconizing reagent (Sigma Aldrich) to reduce non-specific binding. 50 μl of cell protein lysate was incubated with 50 μl of 10 increasing concentrations (2 pM – 1 nM) of [^125^I]apelin-13 in binding buffer containing 50 mM Tris-HCl and 5 mM MgCl2 (Sigma Aldrich), pH 7.4, for 90 minutes at room temperature. 50 μl of 5 μM [Pyr^1^]apelin-13 (Severn Biotech) diluted in binding buffer was used to define non-specific binding. Following incubation, centrifugation (20,000 xg, 10 minutes at 4°C) was used to break equilibrium and pellets washed with Tris-HCl, pH 7.4 at 4°C. Bound radioactivity in cell pellets was counted in a Gamma counter (Packard, COBRA 5003) and data analysed using iterative curve fitting programs EBDA and LIGAND (KELL Package, Biosoft, UK).

**ELISA**

Conditioned supernatant was collected from hESCs and hESC-CMs and apelin and ELA peptide production determined using either Apelin-12 (Human, Rat, Mouse, Bovine) EIA or [pGlu1]-ELA-32 (Human) EIA sandwich ELISA kits according to manufacturer’s protocol (Phoenix Pharmaceuticals). Fresh hESC and hESC-CM was also assayed as a control.

**Immunocytochemistry**

For staining with cardiac markers (Troponin T and α-actinin), cells were washed with PBS and fixed with 4% PFA (ThermoFisher) at RT for 20 minutes. Blocking was performed by incubating with 3% BSA in PBS for 1 hour at RT. For permeabilisation, 0.5% Triton X-100 (Sigma Aldrich) was included during the blocking step. Primary antibody diluted in 3% BSA plus 0.5% Triton-X was added at the desired concentration (Table S4) and incubated at 4°C overnight. Following incubation, cells were washed three times with PBS and then secondary antibody added, diluted to the desired concentration in PBS containing 3% BSA plus 0.5% Triton-X (Table S4). DAPI nuclear stain (ThermoFisher) was also included at 1:10,000 and incubated for 1 hour at RT in the dark. Cells were then subject to a further three washes, PBS added and imaging performed using either the Zeiss LSM 700 confocal microscope or the EVOS FL Cell Imaging System (Invitrogen).

For staining with apelin receptor antibody, wild-type and apelin receptor knockdown hESC-CMs were washed with PBS and fixed with 4% PFA (ThermoFisher) at RT for 20 minutes. Non-specific staining was blocked with PBS + 10% donkey sera + 1% Tween-20 for 2 hours at RT. Primary antibody raised against the apelin receptor (abcam; ab84296), or IgG isotype control (abcam; ab37415), prepared at 1:50 in PBS + 1 % donkey sera + 0.1 % Tween-20 + 3.3 mg/mL bovine serum albumin were added and incubated overnight at 4ºC. Following incubation, cells were washed three times with PBS and then secondary antibody (donkey anti-rabbit Alexa Fluor 488, ab150061), prepared at 1:200 in PBS + 1 % donkey sera + 0.1 % Tween-20 + 3.3 mg/mL bovine serum albumin was added and incubated for 1 hour at RT. Cells were washed three times with PBS before treatment with 10 μg/mL Hoechst 33342 nuclear stain (H3570; Invitrogen) in PBS. Cells were washed a final three times with PBS before maintenance in 100 µL PBS for imaging on the Opera Phenix. Cells were imaged using a 40x/NA1.1 water immersion objective. A blue channel, with excitation of 405 nm and emission filter of 435-480 nm for Hoechst 33342 nuclear marker, and a green channel, with excitation of 488 nm and emission filter of 500-550 nm for Alexa Fluor 488 were used, both at an excitation laser intensity of 50% with a 50 ms exposure time.

**Supplementary Table 4:** Antibodies used for immunocytochemistry.

| **Protein** | **Species** | **Manufacturer (Cat#)** | **Concentration** |
| --- | --- | --- | --- |
| Primary Antibodies | | | |
| Apelin Receptor | Rabbit | Abcam (ab84296) | 1:50 |
| Troponin T | Goat | Abcam (ab64623) | 1:200 |
| α-Actinin | Mouse | Abcam (ab9465) | 1:200 |
| Secondary Antibodies | | | |
| anti-rabbit Alexa Fluor 488 | Goat | ThermoFisher (A11034) | 1:200 |
| anti-goat Alexa Fluor 488 | Donkey | ThermoFisher (A11054) | 1:400 |
| anti-mouse Alexa Fluor 488 | Goat | ThermoFisher (A21121) | 1:400 |

**Flow cytometry**

hESC-CMs were detached using TryPLE Express, collected and pelted by centrifugation. Pellets were resuspended in PBE (PBS supplemented with 0.1% BSA and 2 mM EDTA (Invitrogen)) containing CD90 (Thy-1) Monoclonal Antibody (eBio5E10 (5E10)), PE, eBioscience™ (Invitrogen, 12-0909-42) diluted at 1:50, and incubated at 4°C for 1 hour. PBE washes were performed and cells fixed by resuspending in Fixation/Solubilization solution (BD Cytofix/Cytoperm Fixation/Permeabilization Kit, BD Biosciences) for 20 minutes at 4°C. Cells were washed with 1X BD Perm/Wash Buffer (BD Biosciences), before incubating with PBE containing 1:50 Anti-Cardiac Troponin T-APC antibody (Miltenyi Biotec, 130-120-54300) for 2 hours at 4°C. Final washes were performed, cells resuspended in PBE and run on the LSRFortessa Cell Analyzer (BD Biosciences). Analysis was performed using FlowJo V10.8.1 software (BD Biosciences, NJ, USA).

**Generation of sOPTiKD apelin receptor knockdown system**

The single-step optimised inducible knockdown system (sOPTiKD) was used to generate an apelin receptor tetracycline inducible short hairpin RNA (shRNA) knockdown system as described previously^26^. The pAAV-Puro_siKD targeting vector was a gift from Ludovic Vallier (Addgene plasmid #86695; http://n2t.net/addgene:86695; RRID:Addgene_86695).

shRNA Oligonucleotide Design, Annealing, and Ligation and Plasmid Screening

Known shRNAs targeting the apelin receptor or control beta-2-microglobulin (B2M) gene were identified from the MISSION shRNA TRC database (Sigma Aldrich, Table S5) and modified to include overhangs for insertion into the pAAV-Puro_siKD vector. The U6 promoter requires a starting Guanine, hence if not already present on the shRNA this was added.

**Supplementary Table 5:** Sequences of shRNAs targeting the apelin receptor gene (Sigma Aldrich MISSION shRNA TRC Database).

| **shRNA** | **Sequence** |
| --- | --- |
| APLNR | CCGGGAGAACAGATGCACGAGAAATCTCGAGATTTCTCGTGCATCTGTTCTCTTTTTG |
| B2M | GATCCCGGACTGGTCTTTCTATCTCTTCAAGAGAGAGATAGAAAGACCAGTCCTTTTTTG |

shRNA oligonucleotide annealing and ligation into pAAV-Puro_siKD vector was performed as described by Bertero *et al.*^26^. Ligated products were then transformed into α-Select Gold Efficiency Chemically Competent Cells (Bioline) according to manufacturer’s protocol. Cells were grown overnight at 37°C on ampicillin (Sigma Aldrich) Lysogeny broth (LB, Sigma Aldrich)-agar (Fluka) plates. Overnight liquid culture in 5ml LB + ampicillin shaking at 37°C for individual colonies was carried out, followed by pelleting of 4.5ml bacterial culture by centrifugation. Plasmid DNA was extracted using the GenElute Plasmid Miniprep Kit (Sigma Aldrich) as described previously^26^. 1 ng of purified plasmid DNA was amplified using the LongAmp Taq DNA Polymerase PCR protocol (New England BioLabs) at an annealing temperature of 60°C to assess successful shRNA oligonucleotide insertion using primers AAVSeqF (CGAACGCTGACGTCATCAACC) and AAVSeqR (GGGCTATGAACTAATGACCCCG) as described previously^26^. Control uncut parent plasmid and PCR products were run on a 1.5% agarose gel, with successful shRNA insertion evidenced as a band present at ~390bp, compared to the noticeably smaller empty vector (~295bp). To confirm insertion of the correct oligonucleotide, Sanger Sequencing (Source Biosciences) was performed on amplified product from positive clones. Clones confirmed to be correct were then regrown in 100 ml liquid culture overnight at 37°C, and plasmids purified the following day with the QIAGEN Plasmid Plus Midi Kit following manufacturer’s instructions.

hESC Transfection

To prepare H9 hESCs in 6-well plates for transfection, media was aspirated and cells were washed once with PBS then incubated at 37°C in 1 ml Opti-MEM (ThermoFisher). During incubation, mixes were prepared for transfection by adding 10 µl Lipofectamine 2000 (ThermoFisher) to 240 µl Opti-MEM (mixture A) per well. Alongside this, 4 µg of DNA made up of equal parts of the targeting shRNA vector and two AAVS1 zinc finger nuclease plasmids^26^ was added to 250 μl/well Opti-MEM (mixture B). Mixture A and B were incubated individually for 5 minutes at room temperature, before combining 250 µl of each mixture per well and incubating at room temperature for 20 minutes. Next, 500 µl of the combined mixture was added in a dropwise manner to each well. Following overnight incubation at 37°C, transfection mix was removed, cells washed and media replaced with H9 maintenance media, with daily media change. Once cells had reached 80% confluence, antibiotic selection was initiated by culturing cells in the presence of 1 μg/ml puromycin (Sigma Aldrich), with continued daily refreshment of media. Resistant surviving colonies were mechanically selected and allowed to expand clonally, maintained as detailed above in the presence of puromycin (1 μg/ml).

shAPLNR Genotyping

To determine site-specific targeting of the vector, clonal cells were collected and pelleted by centrifugation at 8,000 xg for 3 minutes. The GenElute Mammalian Genomic DNA Miniprep Kit (Sigma Aldrich) was used according to manufacturer’s instructions and 100 ng of genomic DNA used for each genotyping reaction. Different combinations of primers were used to perform three reactions (detailed in Table S6, all Sigma Aldrich) using the LongAmp Taq DNA Polymerase PCR protocol. PCR products were run on 1% agarose gels, with successfully targeted clones determined according to band pattern^26^.

**Supplementary Table 6:** Primer locations and sequences and PCR conditions used for genotyping of targeted hESCs.

| **PCR Type** | **Primer Location** | **Primer Sequence** | **Amplicon wild-type (bp)** | **Amplicon for transgene insertion** | **Annealing temp. (°C)** | **Extension time** | **Result?** |
| --- | --- | --- | --- | --- | --- | --- | --- |
| **LOCUS** | Genomic; 5' to 5'-HAR | CTGTTTCCCCTTCCCAGGCAGGTCC | 1692 | No band for homozygous targeting, faint band for heterozygous | 65 | > 3 min | Has vector inserted in genomic locus? |
|  | Genomic; 3' to 3'-HAR | TGCAGGGGAACGGGGCTCAGTCTGA |  |  |  |  |  |
| **5’-INT** | Genomic; 5' to 5'-HAR | CTGTTTCCCCTTCCCAGGCAGGTCC | No band | 1103 | 65 | 1 min 30 | Has the correct vector integrated? |
|  | Puromycin | TCGTCGCGGGTGGCGAGGCGCACCG |  |  |  |  |  |
| **3’-INT** | OPTtetR | CCACCGAGAAGCAGTACGAG | No band | 1447 | 60 | 1 min 30 | Has the correct vector integrated? |
|  | Genomic; 3' to 3'-HAR | TGCAGGGGAACGGGGCTCAGTCTGA |  |  |  |  |  |

**RNA-sequencing**

RNA-sequencing data is available through the European Nucleotide Archive (EMBL-EBI) Accession number: PRJEB49219 Title: Apelin receptor knockdown in hESC-cardiomyocytes (embargoed until accepted for publication).

RNA extraction

RNA was extracted using TRIzol reagent (Invitrogen) following the recommended protocol. Cell pellets were thoroughly resuspended in 400 µl TRIzol reagent and incubated for 5 minutes at RT, before adding 80 µl of chloroform (Macron Fine Chemicals). Samples were mixed and incubated for a further 3 minutes before centrifuging for 15 minutes (12,000 xg, 4°C) to induce phase separation. The upper aqueous phase was transferred to a clean Eppendorf tube and 200 µl isopropanol (Sigma Aldrich) added for RNA precipitation. After 10 minutes incubation at RT, samples were centrifuged (12,000 xg, 10 minutes, 4°C), supernatant discarded and pellets air dried at RT for 10 minutes. Elution was performed by adding 20 µl RNase free-water and incubating at 55°C for 15 minutes, with sample RNA concentration determined using a NanoDrop One (ThermoFisher).

RNA processing and sequencing

*Quality control:* The TapeStation RNA ScreenTape (Agilent) was used to assess RNA integrity, generating an RNA Integrity Number (RIN^e^). All samples had RIN^e^ 7.4 – 8.2 (7.74±0.25) which passed QC. This service was performed at Cambridge Genomics Services (Department of Pathology, University of Cambridge).

*Ribosomal RNA removal:* The NEBNext rRNA Depletion Kit (Human/Mouse/Rat) (New England BioLabs) was used following recommended protocol to remove ribosomal RNA, with 6 µl total RNA used per sample.

*Total stranded RNA-sequencing library preparation:* The CORALL Total RNA-Seq Library Prep Kit (Lexogen) was using to generate total stranded RNA-sequencing libraries, with 15 PCR cycles used for the final amplification step. The kit was used as per manufacturer’s instructions apart from AMPure XP PCR Purification beads (Beckman Coulter) and 80% nuclease-free ethanol were used at 1.8 X volume for all purification steps, except the final amplification stage where 1 X volume was used.

Samples were then sent to the Babraham Institute Next Generation Sequencing facility. First, quality control was performed using a 2100 Bioanalyzer (Agilent) to ensure removal of primer and adaptor dimers (120130 bp) and that the majority of fragments were in the size range 200 – 600 bp. Samples were then sequenced using the H9Seq2500 (Illumina) as 100 bp Single-End sequencing runs, with 15 RNA-seq libraries sequenced per lane.

Data analysis

*Processing of next generation sequencing data:* First, fastqc analysis was performed on next generation sequencing fastq output files, carrying out basic quality checks and aligning to reference genomes (<https://www.bioinformatics.babraham.ac.uk/projects/fastqc/>). Trim Galore! was used to trim reads based on Phred quality score, with a score of 20 used as a threshold correlating with an error rate of less than 1 in 100 bases and a trimming rate of less than 0.1^51^ (<https://www.bioinformatics.babraham.ac.uk/projects/trim_galore/>). HiSAT2 was then used to align data to the Homo sapiens reference genome, GRCh38/hg38 (<http://daehwankimlab.github.io/hisat2/>). These steps were performed by Simon Andrews and Felix Krueger at the Babraham Institute Bioinformatics Group. Trimmed and aligned sequencing files were analysed in SeqMonk (v1.42.0) by importing as BAM files (<http://www.bioinformatics.babraham.ac.uk/projects/seqmonk/>).

*Differential gene expression analysis:* Read count quantitation was performed and global normalisation carried out relative to total read count for each replicate, with data expressed as reads per million (RPM). Differential gene expression analysis was performed using DESeq2^52^. Non-log transformed raw read counts were inputted and global normalisation performed to total library size. RPM values ≥ 1.0 were deemed to be above noise. Benjamini-Hochberg correction for multiple testing was used, with false discovery rate of 5 % was deemed acceptable. This identified 272 up- or downregulated differentially expressed genes.

Human tissue samples

Surgical samples of control human heart tissue that were not suitable for transplantation were obtained from Royal Papworth Hospital Research Tissue Bank with informed consent and ethical approval (05/Q104/142). Tissues were snap frozen in liquid nitrogen before storage at -80°C. RNA extraction was performed as described above using TRIzol reagent.

**Voltage and calcium imaging**

To assess voltage signalling, hESC-CMs were loaded with FluoVolt Membrane Potential voltage sensitive dye (ThermoFisher) diluted 1:1000 in Tyrode’s solution (5 mM glucose, pH7.4), plus PowerLoad solution diluted 1:100. For calcium signalling Fluo-4, AM (Invitrogen) was diluted 1:1000 in CDM-BSA. For both dyes, media was aspirated from cells and replaced with dye solution, before incubating at 37°C for 30 minutes. Dye was then aspirated and replaced with Tyrode’s solution. Cells were imaged using the Axio Observer A1 Inverted Phase Contrast Fluorescence Microscope (Zeiss) with LabCam adaptor mounted and videos recorded using an iPhone 7. Cells were paced at 1Hz using the C-Pace EM fitted with 6-well plate adaptor (IonOptix). Generated videos were loaded into a custom MATLAB (R2021a, MathWorks, Natick, MA, USA) code designed to extract values for time to peak and decay time.

**Engineered heart tissue generation**

3D engineered heart tissues (EHTs) were produced as described previously^27,28^ from hESC-CMs generated as described above. hESC-CMs were cast alongside HS-27A cells in a collagen gel matrix (Table S7) in preprepared polydimethylsiloxane (PDMS, Dow) moulds treated with 5% pluronic acid F127^27^ (Sigma Aldrich). Cast constructs were incubated at 37°C for 30 minutes to allow solidification, and then 6ml RPMI media (ThermoFisher) supplemented with B27 plus insulin (ThermoFisher) added. EHTs were cultured for 14 days, with RPMI refreshed every second day and tetracycline included at a concentration of 1 µg/ml to induce apelin receptor knockdown. Spontaneous contraction was observed within 2-5 days.

| **Component** | **Volume/ml (µl)** | **Supplier** |
| --- | --- | --- |
| 10x RPMI-1640 medium (Gibco) | 85.75 | Sigma Aldrich |
| 1M NaOH | 12.60 | Fisher Scientific |
| Geltrex (Invitrogen) | 142.91 | Fisher Scientific |
| Collagen I Rat Protein (Gibco) | 541.77 | ThermoFisher |
| Sterile mH_2_0 | 150.00 | - |
| HEPES buffer | 20 | Sigma Aldrich |
| Additional NaOH | N (dependent on visualised colour change) | Fisher Scientific |
| Additional sterile mH_2_0 | 46.97 – N | - |

**Supplementary Table 7:** Components of collagen gel used for casting 3D EHTs.

**Aurora force transducer measurements**

EHTs were subjected to force measurements by removing from their moulds and attaching them between a force transducer (model 400A, Aurora Scientific) and length controller (model 312B, Aurora Scientific), bathed in Tyrode’s solution at 37°C. Additive strain was applied, with the length controller stretching the constructs in 4% intervals from resting length to 24% strain. Spontaneous and paced (1, 1.5 and 2 Hz, 5 V and 50 ms pulse duration) contraction was recorded using LabView software, and output analysed using a custom MATLAB code.

**Collagen imaging**

EHTs were fixed with 4% paraformaldehyde before incubating overnight in 30% sucrose (Fisher Scientific) solution. Tissues were then cryoembedded and sectioned, and glass coverslips mounted using Permanent Aqueous Mounting Media (Bio Rad). A Zeiss LSM880 multiphoton system, with a Newport Spectraphysics Insight DS+ laser was used to perform second-harmonic imaging microscopy (SHIM), allowing visualisation of collagen without exogenous staining. SHIM images were acquired using a 10x 0.5NA water dipping objective, excitation at 920 nm and detection at 437-463 nm.

**Aniline blue collagen staining**

Wild-type and apelin receptor knockdown EHTs were fixed with 4% paraformaldehyde before incubating overnight in 30% sucrose (Fisher Scientific) solution. Tissues were then cryoembedded and sectioned onto microscopy slides before storage at -70 °C until use. On the day of use, slide mounted sections were thawed for 20 mins at room temperature. Sections were then stained with a ready-to-use ‘Trichrome Stains (Masson)’ kit from Sigma Aldrich, in accordance with the manufacturer’s protocol. In brief, slide mounted sections were treated with Bouin’s solution for 15 mins at 56 °C in Hellendahl jars before cooling and washing in running tap water for 5 mins. Slides were then treated with working phosphotungstic / phosphomolybdic acid solution for 5 mins, before treatment with aniline blue solution for 5 mins to stain collagen fibres. Note that Weigert’s iron hematoxylin and Biebrich’s scarlet-acid fuschin solutions, which stain nuclei and cytoplasm respectively, were not included, as they made interpretation of qualitative results more difficult. The exclusion of these solutions has been reported previously as having no effect on the staining with aniline blue^53,54^. Subsequently, slides were differentiated in 1% acetic acid for 30 seconds. Slides were rinsed with tap water before dehydrating through alcohol (70%, 95%, 100%, 100%), and then cleared with xylene for 1 h. Sections were mounted with aqueous mounting solution provided in the kit and covered with a coverslip. Bright-field images of aniline blue stained sections were acquired using a Zeiss Axio Scan Z1 slide scanner with a Plan-Apochromat 20x/NA0.8 M27 objective lens connected to a Hamamatsu Orca Flash camera. All images were acquired using ZEN software (Zeiss) and were visualised and analysed using Orbit Image Analysis (ORBIT) software.

**Data analysis and statistics**

All data are represented as mean±sem. The n values are stated in the figure legends. Statistical unpaired, two-tailed Student’s t-tests or one way ANOVA followed by Tukey’s post hoc test were performed and a p value <0.05 considered as significant. Graphical presentation and statistical analyses were performed using GraphPad Prism v7.05 (GraphPad Software, La Jolla, CA, USA) unless otherwise stated. Independent replicates for hESCs are defined as cells from distinct passages and for cardiomyocytes are defined as cells generated from distinct differentiations.

**Supplementary References**

51. MacManes MD. On the optimal trimming of high-throughput mRNA sequence data. *Front Genet.* 2014;**5**:13.

52. Love MI, Huber, W, Ander, S. Moderated estimation of fold change and dispersion for RNA-seq data with DESeq2. *Genome Biol.* 2014;**15**:550.

53. Saxena R. Dual Immunohistochemistry-Aniline Blue Stain: The Trichrome Stain Revisited. *Journal of Histotechnology* 2010;**33**:25-29

54. Rieppo L, Janssen L, Rahunen K, Lehenkari P, Finnila MAJ, Saarakkala S. Histochemical quantification of collagen content in articular cartilage. *PLOS One* 2019;**14**: e0224839.
